# Exploration and Development of PPAR Modulators in Health and Disease: An Update of Clinical Evidence

**DOI:** 10.3390/ijms20205055

**Published:** 2019-10-11

**Authors:** Hong Sheng Cheng, Wei Ren Tan, Zun Siong Low, Charlie Marvalim, Justin Yin Hao Lee, Nguan Soon Tan

**Affiliations:** 1 School of Biological Sciences, Nanyang Technological University Singapore, 60 Nanyang Drive, Singapore 637551, Singapore; 2 Lee Kong Chian School of Medicine, Nanyang Technological University Singapore, 11 Mandalay Road, Singapore 308232, Singapore

**Keywords:** clinical trials, metabolic syndrome, type 2 diabetes mellitus, cancer, non-alcoholic fatty liver diseases, cardiovascular diseases, neurological disorders

## Abstract

Peroxisome proliferator-activated receptors (PPARs) are nuclear receptors that govern the expression of genes responsible for energy metabolism, cellular development, and differentiation. Their crucial biological roles dictate the significance of PPAR-targeting synthetic ligands in medical research and drug discovery. Clinical implications of PPAR agonists span across a wide range of health conditions, including metabolic diseases, chronic inflammatory diseases, infections, autoimmune diseases, neurological and psychiatric disorders, and malignancies. In this review we aim to consolidate existing clinical evidence of PPAR modulators, highlighting their clinical prospects and challenges. Findings from clinical trials revealed that different agonists of the same PPAR subtype could present different safety profiles and clinical outcomes in a disease-dependent manner. Pemafibrate, due to its high selectivity, is likely to replace other PPARα agonists for dyslipidemia and cardiovascular diseases. PPARγ agonist pioglitazone showed tremendous promises in many non-metabolic disorders like chronic kidney disease, depression, inflammation, and autoimmune diseases. The clinical niche of PPARβ/δ agonists is less well-explored. Interestingly, dual- or pan-PPAR agonists, namely chiglitazar, saroglitazar, elafibranor, and lanifibranor, are gaining momentum with their optimistic outcomes in many diseases including type 2 diabetes, dyslipidemia, non-alcoholic fatty liver disease, and primary biliary cholangitis. Notably, the preclinical and clinical development for PPAR antagonists remains unacceptably deficient. We anticipate the future design of better PPAR modulators with minimal off-target effects, high selectivity, superior bioavailability, and pharmacokinetics. This will open new possibilities for PPAR ligands in medicine.

## 1. Introduction

Peroxisome proliferator-activated receptors (PPARs) are members of the nuclear receptor superfamily whose physiological functions are linked to metabolism, energy homeostasis, cellular development, and differentiation. Three members of PPARs have been identified, namely PPARα, PPARγ, and PPARβ/δ. Upon ligand binding, PPARs translocate into the nucleus, where they heterodimerize with retinoid X receptor and bind to peroxisome proliferator response elements (PPREs) to regulate the transcription of target genes [1]. Despite sharing a high degree of structural homology, the three PPAR isoforms have distinct functional roles, tissue distribution, and ligand-binding properties [2]. The characteristics of human PPARα, β/δ, and γ are well-reviewed [3,4,5] and will not be elaborated herein.

**Figure 1:**
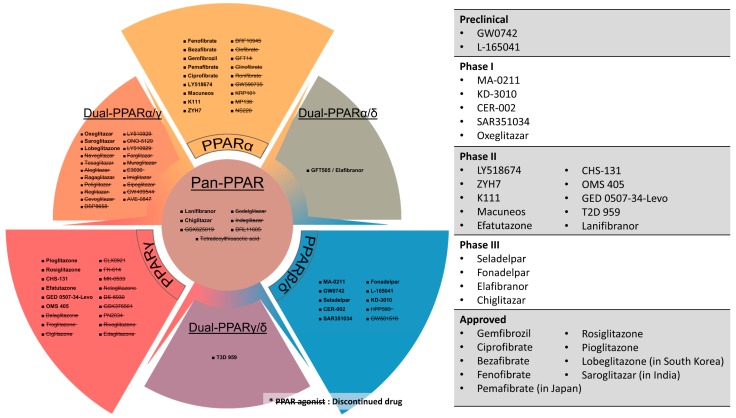
PPAR agonists, their PPAR target(s) and current status in clinical pipeline. Drugs with strikethrough mark (e.g., PPAR agonist) have been discontinued at clinical or preclinical stages.

Owing to their crucial metabolic regulatory roles and excellent druggability, many PPAR agonists have been synthesized for the treatment of metabolic diseases, especially dyslipidemia and type 2 diabetes mellitus (T2DM). For instance, fibrates which are selective PPARα agonists, are often used in combination with statins to treat atherogenic hyperlipidemia and hypertriglyceridemia [6]. Likewise, thiazolidinediones (TZDs), which are potent PPARγ activators, are used as insulin sensitizers to manage T2DM patients [7]. The clinical success of fibrates and TZDs have not only propelled the development of various PPARα or γ agonists but also sparked the creation of novel PPAR modulators including selective PPARβ/δ activators, dual-PPAR agonists, and pan-PPAR agonists [2]. Aside from dyslipidemia and T2DM, PPARs also have profound implications on other facets of metabolic syndrome (MetS), like diabetic complications, non-alcoholic fatty liver disease (NAFLD), as well as non-metabolic disorders including neurodegenerative diseases, cancers, and inflammatory diseases. As a result, the clinical benefits of PPAR agonists have been assessed in a wide variety of diseases and health complications [8]. Different PPAR agonists and their current clinical statuses are illustrated in Figure 1.

Undeniably, drugs that target PPARs are of paramount scientific and clinical significance. In the review, we aim to consolidate existing clinical evidence of PPAR agonists and antagonists to highlight their effectiveness, health benefits, clinical prospects, and developmental challenges. Since the lipid-lowering activity of PPARα agonists and insulin-sensitizing effect of PPARγ agonists are extremely well-established and have been widely exploited to improve dyslipidemia and T2DM [9,10,11,12], these aspects will be excluded from this review. Extra emphasis will be placed on new classes of PPAR modulators like dual- and pan-PPAR agonists which are collectively known as “glitazars”. Essentially, this review will provide a comprehensive and up-to-date overview of the latest development of PPAR modulators for the treatment of various diseases based on existing clinical data.

## 2. Mechanistic Rationales for Targeting PPARs in Various Human Diseases

PPARs are critical lipid sensors and regulators because of their indispensable roles in various lipid-related bioactivities such as lipid transport, adipocyte differentiation as well as the metabolism of various lipid components like fatty acids, ketone bodies, triglycerides, and cholesterols. Hepatic PPARα stimulates fatty acid catabolism by modulating the expression of lipoprotein lipase (*LPL*), apolipoprotein genes (*APOA1*, *APOA2*, and *APOA5*), fatty acid transport and oxidation genes (*FABP1*, *FABP3*, *ACS*, *ACO*, *CPT1*, and *CPT2*), as well as genes for HDL metabolism (*PLTP*) and ketone synthesis (*HMGCS2*) [13,14]. As a result, hepatic PPARα activation is associated with substantial triglyceride clearance and increased plasma HDL level, underpinning the clinical use of PPARα agonists to treat hyperlipidemia and cardiovascular disease (CVD). On the other hand, PPARγ selectively promotes lipid uptake and lipogenesis in the adipose tissues, leading to lowered circulating triglycerides and free fatty acids, and insulin resistance [15]. Furthermore, in the adipocytes, genes responsible for insulin-dependent glucose uptake (*GLUT4*, *IRS-1*, *IRS-2*, and c-Cbl associated protein) and adipokines (adiponectin, resistin, leptin, and tumor necrosis factor-α) are also PPARγ responsive [15]. These adipokines can influence insulin signaling. Consequently, PPARγ activation in adipocytes is sufficient to enhance systemic insulin sensitivity, making PPARγ agonists a potent antidiabetic agent [16]. In contrast to PPARα and PPARγ, much less is known about the regulatory mechanism of PPARβ/δ. Aside from driving fatty acid catabolism and energy uncoupling, activation of PPARβ/δ has been demonstrated to favor β-oxidation over glycolysis in the skeletal muscles, which dramatically enhanced muscle endurance to physical exercises [17]. The stimulatory effect of PPARβ/δ on fatty acid oxidation and mitochondrial activity may help to preserve pancreatic β-cell function and insulin secretion in the event of prolonged lipotoxicity [18]. In essence, all three PPARs occupy pivotal niches in energy metabolism, rendering their agonists among the most extensively tested drugs for diseases associated with MetS, such as prediabetes, T2DM, obesity, CVD, and atherogenic dyslipidemia, as well as endocrine diseases like polycystic ovarian syndrome (PCOS). Furthermore, the close link between PPARs, metabolism and liver functions also underscores PPARs as potential targets for the liver manifestations: non-alcoholic fatty liver disease (NAFLD), non-alcoholic steatohepatitis (NASH), and primary biliary cholangitis (PBC). Owing to the distinct roles of different PPARs, concomitant activation of multiple PPARs is believed to elicit a superior therapeutic efficacy. Such a speculation leads to the creation of dual- and pan-PPAR agonists which act on two or all PPAR isoforms. The new classes of PPAR modulators are actively being investigated for CVD, T2DM, dyslipidemia, NASH, PBC, and MetS.

There is increasing evidence showing that the cardiovascular benefits of PPAR agonists are attributed, at least in part, to the activation of the endothelial nitric oxide synthase (eNOS) [19]. Under physiological conditions, nitric oxide produced by eNOS acts as a vasodilator and anti-thrombotic agent to safeguard endothelial functions. The activity of eNOS is significantly compromised in CVD and atherosclerosis, resulting in low nitric oxide bioavailability and the disruption of endothelial vasculature. All three subtypes of PPARs can promote eNOS activation. For example, fibrates can enhance nitric oxide biosynthesis by upregulating eNOS expression, stabilizing eNOS mRNA, and stimulating eNOS activation via PI3K, MAPK, and AMPK pathways [20,21]. PPAR β/δ and PPARγ also modulate eNOS activity through the PI3K-Akt pathway [22,23]. The stimulatory effect of PPARγ on eNOS activation and stability is also facilitated by other intermediates, including heat shock protein 90, adiponectin, and Src homology region 2-containing protein tyrosine phosphatase 2 [24,25,26]. Taken together, the effect of PPARs on eNOS and nitric oxide production lays the groundwork for the clinical use of PPAR agonist in CVD and hypertension.

PPARs have also emerged as important regulators of innate immunity and inflammatory response. They form a crucial link between metabolic disorders and chronic low-grade inflammation, which often co-manifest and inseparably intertwine in chronic metabolic diseases. PPARs modulate inflammatory response via various direct and indirect mechanisms [27]. For example, inflammatory mediators and PPARα ligand, leukotriene B4 can exert a negative feedback mechanism via PPARα activation to limit its activity and to resolve an inflammatory response [28]. PPARα also interferes with the proinflammatory activity of NF-κB by modulating the gene expression of IκB, an NF-κB inhibitor [29]. PPARα interacts with glucocorticoid receptor α or estrogen receptor to transrepress other proinflammatory transcription factors for anti-inflammatory effects [30,31]. Likewise, the transrepression of inflammatory response genes due to ligand-dependent SUMOylation of PPARγ has been observed [32]. Such a post-translation modification of PPARγ reinforces PPARγ-nuclear receptor corepressor-histone deacetylase-3 complexes, thus stabilizing NF-κB in its repressed, promoter-bound state [32]. In dendritic cells, PPARγ takes part in the regulation of various processes like antigen uptake, cellular activation and maturation, cytokine production, and lipid antigen presentation [33]. Furthermore, macrophage PPARγ can inhibit genes encoding proinflammatory molecules while activating the expression of anti-inflammatory mediators to promote anti-inflammatory effect [15,27]. Together with their glucose and lipid regulatory activities, the anti-inflammatory effect of PPARα and γ is beneficial in medical conditions where inflammation is one of the major driving forces of disease exacerbation, such as NASH and atherosclerosis. In hepatitis C infection, insulin resistance and hepatic steatosis appear to benefit viral core protein expression and confer anti-viral drug resistance [34]. Targeting PPARs, which is effective to resolve these abnormalities, can possibly aid in viral and metabolic-related hepatitis. Due to the anti-inflammatory properties, PPARs are also exploited to mitigate acute inflammatory flares in various autoimmune, inflammatory, and infectious diseases, including rheumatoid arthritis, systemic lupus erythematosus (SLE), sepsis, endometriosis, ulcerative colitis, and asthma. Since neuroinflammation has been one of the prominent themes in the pathogenesis for multitudes of neurological diseases, targeting PPARs may also reap beneficial outcome [35,36,37]. Thus, PPAR agonists are prospective stand-alone or co-administered therapeutic drugs for many neurological or neurodegenerative diseases like amyotrophic lateral sclerosis, multiple sclerosis, cognitive impairment, and Alzheimer’s disease as well as mental disorders like depression, addiction, and schizophrenia. Unlike PPARα and γ, a role for PPARβ/δ in inflammation remains controversial. Therefore, the efficacy of PPARβ/δ agonists is rarely examined in inflammatory conditions.

Several PPARs directed processes were linked to either pro- or anti-tumorigenesis. For instance, PPARα inhibits angiogenesis by hindering endothelial cell proliferation, increasing the expression of angiogenic inhibitors like endostatin and thrombospondin 1, and downregulating VEGF and cytochrome P450 CYP2C [38]. The interaction between PPARα and NADPH Oxidase 1 also modulates angiogenesis [39]. On the other hand, ligand-activated PPARγ facilitates terminal differentiation, promotes cell cycle arrest, and apoptosis of cancer cells [40]. PPARγ agonists have been shown to regulate the expression of cell cycle mediators like cyclin D1 and cyclin-dependent kinase inhibitors (p21 and p27), resulting in the attenuation of cell cycle progression and proliferation [41,42,43]. PPARγ agonists also trigger increased apoptotic signaling via the overexpression of pro-apoptotic PTEN, BAX, and BAD, although off-target effects cannot be excluded [44,45]. Moreover, PPARγ also promotes epithelial differentiation and stabilizes the differentiated phenotype by upregulating key proteins like keratins, E-cadherin, alkaline phosphatase, and developmentally-regulated GTP-binding protein 1 [40,46]. The aforementioned anti-tumorigenic properties of PPARα and γ bring about extensive clinical studies that aim to treat malignancy by targeting these nuclear receptors. Again, unlike PPARα and γ, the biological function of PPARβ/δ in tumorigenesis remains complex and conflicting. Current evidence supports an oncogenic tendency for PPARβ/δ activation and thus, has raised a question mark over the clinical development and safety of PPARβ/δ agonists.

**Figure 2:**
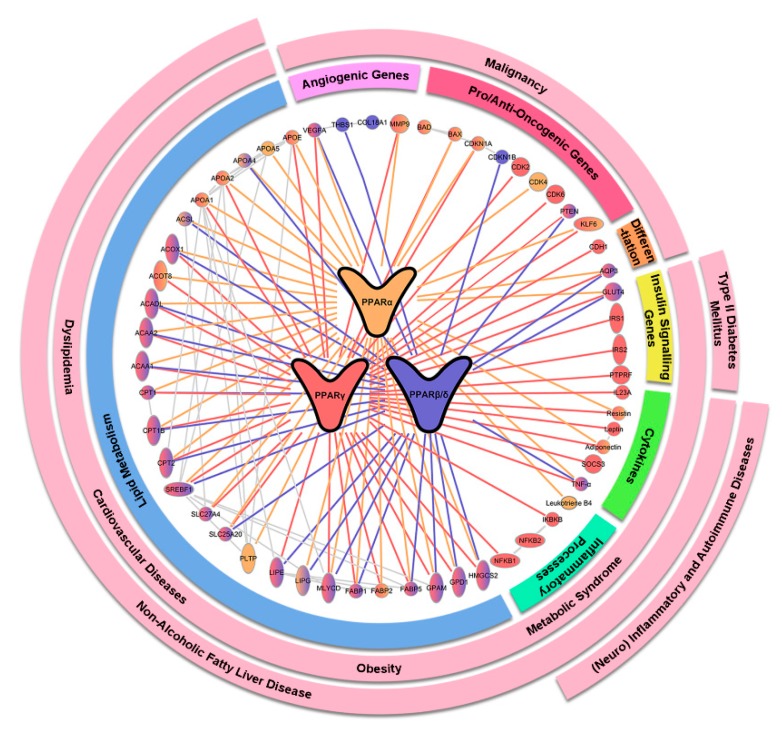
Key target genes and interacting proteins of PPARα, γ, and β/δ and their downstream biological functions and implicated health conditions. Genes and proteins regulated by or interacting with PPARα, γ, and β/δ are represented by yellow, red, and blue lines, respectively. The downstream biological functions of the genes are reflected in the innermost circle while the implicated health conditions are highlighted in pink in outer circles.

Essentially, PPARs are integral in various biological processes in energy metabolism, homeostasis, inflammation, cellular proliferation, and differentiation. The multi-functionality, along with the excellent druggability, makes them an ideal therapeutic target for many health conditions. The PPAR-implicated pathways and related diseases are outlined in Figure 2. Next, we will summarize recent clinical evidence of PPAR agonists in various disorders.

## 3. Type 2 Diabetes Mellitus (T2DM)

The use of PPARγ agonists in T2DM is arguably one of the earliest clinical applications of PPAR agonists that are built upon the discovery and knowledge of PPARs. The clinical efficacy of PPARγ agonists as an oral antidiabetic agent is well-established [47,48,49]. Therefore, such activities and the resultant benefits of both approved and experimental PPARγ agonists, including pioglitazone, rosiglitazone, GSK376501, CHS-131, PN2034, FK614, MK0533, rivoglitazone, and balaglitazone, will not be discussed here.

Despite their clinical feasibility, TZDs have some inherent limitations and side effects [50]. These issues, coupled with the ever-growing prevalence of T2DM, drive the creation of dual- and pan-PPAR agonists with hopes to yield better therapeutic effect and minimize adverse events. This new class of drugs garnering overwhelming enthusiasm from the medical research community. To date, several dual-PPARα/γ agonists have progressed to late-phase clinical trials, including muraglitazar [51,52,53], tesaglitazar [54,55,56,57,58], aleglitazar [59,60,61], lobeglitazone [62,63], and MK0767 [64] (NCT00543010; NCT00543361, NCT00543491, NCT00543517, NCT00543738, NCT00543751, NCT00543816, and NCT00543274). All these dual-PPARα/γ agonists effectively normalize glucose- and lipid-abnormalities in T2DM patients when used as mono- or combined therapy with other glucose-lowering drugs. Nevertheless, most of these drugs have tremendous safety concerns. For example, muraglitazar was approved by the United States Food and Drug Administration in 2005 for its use in controlling blood glucose levels in diabetic patients, but a reanalysis of the data suggested that muraglitazar resulted in an excess incidence of the composite end-point of death, major adverse cardiovascular events and congestive heart failure [65]. Likewise, tesaglitazar and aleglitazar are linked to kidney impairment [66,67,68], whereas MK0767 may be carcinogenic [69]. High risk-to-benefit ratio led to the cessation of further development on muraglitazar, tesaglitazar, aleglitazar, and MK0767. Similarly, other dual-PPARα/γ agonists in early clinical pipeline like naveglitazar (NCT00065312), ONO-5129 (NCT00335712; NCT00212641) and DSP8658 (NCT01042106) also suffered similar setbacks and were no longer in development. At the point of penning down this review, lobeglitazone is still under active development for T2DM (NCT02338921; NCT03770052). Long-term animal studies revealed no carcinogenicity with lobeglitazone [70,71], and a Phase III human trial showed comparable efficacy and adverse events between lobeglitazone and pioglitazone in T2DM patients [63]. In 2013, lobeglitazone had been approved for T2DM in South Korea with the tradename “Duvie” and had since been under post-marketing surveillance. The fact that no severe safety issue arises with the drug provides some forms of reassurance to its safety. In short, the creation of dual-PPARα/γ agonists for T2DM is not a fiasco, but future development needs to ensure a desirable safety profile before clinical testing to regain the faith in this pharmacological class.

Compared to dual-PPARα/γ agonists, dual-PPARα/δ enjoyed greater success in T2DM. Elafibranor (also known as GFT505), which is a dual-PPARα/δ agonist, has shown a potent ameliorative effect on insulin resistance, hyperglycemia and dyslipidemia in obese patients with impaired glucose tolerance without significant safety concern [72,73]. Elafibranor also conferred significant hepatoprotective effect by lowering hepatic lipid deposition and liver enzymes [72]. The unexpected health benefit encouraged the developing company (Genfit) to redirect the research focus of elafibranor towards liver diseases like NAFLD and PBC. Such strategic move successfully placed elafibranor at the forefront of NAFLD drug exploration (see Section 7). Currently, there is no ongoing clinical project on the antidiabetic activity of elafibranor, but its potential in this aspect remains positive.

Several pan-PPAR agonists, including indeglitazar (EUCTR 2005-004227-19; NCT00425919), tetradecylthioacetic acid (NCT00605787), chiglitazar [74,75], and lanifibranor (NCT03459079) have been evaluated for T2DM. Clinical outcomes of most of these drugs are not accessible, but tetradecylthioacetic acid was discontinued due to impaired cardiac performance. The developmental status of indeglitazar is possibly halted, considering that the original developing company (Plexxikon) has been acquired by another pharmaceutical company (Daiichi Sankyo). An exception is chiglitazar which was recently revealed to be well-tolerated and more effective in lowering glycated hemoglobin A1c and restoring insulin sensitivity compared to placebo or sitagliptin based on two Phase III trial [74,75]. With chiglitazar and lanifibranor being active in T2DM clinical pipeline, the prospect of pan-PPAR agonists as antidiabetic agents remains optimistic. Furthermore, given the promising clinical outcomes and good safety profile from chiglitazar trial, future studies are likely to emphasize on the long-term drug efficacy in the prevention of diabetic complications, namely nephropathy, retinopathy, and neuropathy.

Although the finding from FIELD study showed that fenofibrate, a PPARα agonist, has a negligible impact on glycemic control in T2DM patients [76], it did not limit the exploration of fibrates in diabetic complications. In the ACCORD Eye Study, fenofibrate significantly hindered the progression of diabetic retinopathy [77]. A Phase IV trial is currently underway to further validate the retino-protective effect of fenofibrate among T2DM patient (NCT03439345). A separate Phase III trial that aimed to evaluate the efficacy of pemafibrate in diabetic retinopathy was terminated due to subject recruitment issue (NCT03345901). Compared to other synthetic PPAR ligands, the clinical efficacy of PPARβ/δ agonists in T2DM is not well-elaborated. Two Phase II trials were initiated to investigate the effect of GW677954 in diabetic patients (NCT00437164; NCT00196989), but one was prematurely terminated due to carcinogenicity of the drug shown in animal studies. The developing company GlaxoSmithKline has discontinued the PPARβ/δ agonist.

The clinical outcomes of different PPAR agonists in T2DM are summarized in Table 1. Taken together, safety concern remains a considerable obstacle for the clinical development of many novel PPAR agonists, resulting in high attrition of dual- and pan-PPAR agonists as well as PPARβ/δ agonists. However, these setbacks did not stop the exploitation of PPARs for the treatment of T2DM and other diseases. In fact, the inter-drug differences in terms of the side effects suggest that the origin of these unwanted effects is likely off-target reactions that are independent of PPAR. Considering the promising outcomes and good safety profile of chiglitazar, elafibranor, and lobeglitazone, the co-activation of multiple PPARs is still an exciting approach to treat T2DM and its complications.

## 4. Cardiovascular Diseases (CVDs)

As CVDs are the leading cause of death globally, there is tremendous interest to develop more effective treatment strategies. PPAR agonists are potential CVD drugs given that the leading regulatory role of PPARs in metabolism. New development leading this front are dual or pan-PPAR activators due to their synergistic agonistic effect to multiple PPAR receptors. Unfortunately, in a Phase III clinical trial (AleCardio), treatment with aleglitazar—a dual PPARα/γ agonist, failed to modify cardiovascular risk among T2DM patients but, instead, was associated with severe adverse effects, like heart failure, gastrointestinal hemorrhages, and renal dysfunction [78]. The safety concern and clinical futility prompted its developing company Roche to halt clinical activities of aleglitazar, including another larger Phase III trial (AlePrevent), which had also tested aleglitazar’s cardiovascular benefits [79]. Recent post hoc analysis of AleCardio trial data indicates that the concurrent use of clopidogrel (anti-platelet agent) could potentially interfere the metabolism of aleglitazar by inhibiting CYP2C8, subsequently prolonging its clearance and amplifying the toxicity [80]. This finding may serve as supportive evidence to revive the clinical development of aleglitazar, given its outstanding efficacy shown in a Phase II trial [60].

The lipid-lowering effect of PPARα agonists justifies their clinical development for CVD. As the first fibrate, clofibrate was trialed for CVD in the 1960s, the result of which was disappointing because it did not reduce the incidence of fatal heart attacks and angina, and was linked to the increased onset of gallstones and cholecystectomies [81]. A long-term follow-up study revealed that individuals who had previously exposed to clofibrate and stopped had higher mortality compared to the placebo group [82]. The clinical use of clofibrate has been discontinued. Unlike clofibrate, other PPARα agonists have been shown to reduce CVD incidence. For instance, bezafibrate improved lipid profile and reduced fibrinogen by 18%, all-cause mortality by 10%, and non-fatal coronary events by 40% among patients with T2DM, dyslipidemia or existing CVD complications [83,84,85,86]. Likewise, the cardiovascular benefits of fenofibrate and gemfibrozil have been repeatedly proven in many clinical trials [76,87,88,89]. In fact, a recent systematic review concluded that fibrates significantly lower CVD events and myocardial infarction by 16% and 21% respectively among subjects without existing CVD issues, strongly pointing out the clinical significance of PPARα agonists as an effective primary preventive therapy against CVD [90]. Currently, the efficacy of a new PPARα agonist modulator—pemafibrate in CVD is under clinical testing (CTRI/2017/07/009172). In essence, the beneficial effects of PPARα agonists in CVD prevention are widely accepted. Nonetheless, it is worth mentioning that their clinical use is often overshadowed by another class of lipid-lowering drugs—statins—which have been shown to exhibit similar, if not superior efficacy compared to fibrates.

The cardioprotective effect of TZDs has also been observed clinically [91]. In diabetic patients, pioglitazone improved myocardial glucose uptake and myocardial perfusion by 75% and 16% in addition to the enhanced diastolic and systolic function of the heart [92]. The PROactive study concluded that pioglitazone was beneficial in reducing cardiovascular endpoints, namely cardiovascular death, stroke, non-fatal myocardial infarction, and acute coronary syndrome among

T2DM patients [93,94,95]. The IRIS trial discovered that the cardioprotective effect of pioglitazone was considerable even in prediabetes patients, whereby a 24% risk reduction in stroke and myocardial infarction was observed among those treated with pioglitazone compared to placebo [96]. Moreover, in patients who had undergone percutaneous coronary intervention, pioglitazone effectively suppressed in-stent neointimal hyperplasia besides reducing restenosis and incidence of target lesion revascularization [97,98,99,100]. Compared to sulfonylurea, the therapeutic effect of pioglitazone in delaying atherosclerotic progression is superior as exemplified by the lower carotid artery intima-media thickness and atheroma volume [101,102,103]. Although conflicting results about pioglitazone efficacy have been reported in some studies [104,105,106,107], a recent meta-analyses of ten randomized control trials (RCTs) reinforced the finding that the treatment with pioglitazone can lower the risk for major cardiovascular events by approximately 26% [108]. Unlike pioglitazone, the therapeutic effect of rosiglitazone on CVD is modest at best [109,110]. In fact, several meta-analyses have also reported a significant causative relationship between rosiglitazone use with increased risk of heart failure, myocardial infarction, and other major cardiovascular events, a finding which raised substantial controversy about its clinical application [111,112,113].

The clinical outcomes of different PPAR agonists in CVD are summarized in Table 2. Overall, in terms of CVD onset, PPARα agonists generally exhibit a remarkable preventive effect. Pioglitazone, but not rosiglitazone, maybe an excellent drug for primary and secondary prevention of CVD events in T2DM patients, with possible application in those with prediabetes. However, using TZDs as CVD preventive medicines in non-diabetic patients is strongly discouraged considering their adverse effects. The clinical development of dual-PPAR agonist in CVD has ceased.

## 5. Dyslipidemia

Fibrates are PPARα agonists which have been long-established as a lipid-lowering agent. Several meta-analyses have demonstrated that fibrates can reduce total cholesterol and triglycerides while increasing high-density cholesterol [10,114], subsequently lowering cardiovascular risk among patients with atherogenic dyslipidemia [9,115,116]. Therefore, we will not discuss the clinical efficacy of both approved (fenofibrate, bezafibrate, gemfibrozil) and investigational PPARα agonists (LY518674, ZYH7, GW590735, K-111) in dyslipidemia. An exception is pemafibrate, which was approved in Japan in 2017 for the treatment of hyperlipidemia. Pemafibrate is marketed as a selective PPARα modulator due to its superior selectivity for PPARα and markedly higher potency (>2500-fold) compared to fenofibrate [117]. In Phase III clinical trials, pemafibrate significantly reduced the circulating triglycerides by more than 45% after 12–24 weeks of treatment [118,119]. The reduction of triglycerides was comparable to fenofibrate at 200 mg/day, but with fewer incidences of adverse effects [118,119]. In patients with T2DM and hypertriglyceridemia, pemafibrate also decreased other lipid components, namely non-HDL and remnant lipoprotein cholesterols, apolipoprotein (Apo) B100, ApoB48, ApoCIII while enhanced insulin sensitivity score, HDL-cholesterols, and ApoA-I levels in the blood circulation [120]. In dyslipidemic patients with CKD, pemafibrate did not adversely affect the kidney function [121]. Thus, given the favorable safety profile and comparable performance to other fibrates, pemafibrate is a superior PPARα agonist for dyslipidemia.

The lipid-lowering effect of TZDs has been observed in diabetic patients, suggesting a role as a lipid-lowering agent. However, according to Slim et al. (2011), treatment with rosiglitazone for 12 weeks failed to improve hypertriglyceridemia in individuals without diabetes, implying that the hypolipidemic effect is dependent on its insulin-sensitizing properties [122]. As a result, using TZD to treat non-diabetic dyslipidemic patients is not recommended. On the other hand, two PPARβ/δ agonists, namely GW501516 and seladelpar (alternatively known as MBX-8025) have been clinically tested to assess their lipid-lowering activities. Both investigational drugs promote a favorable lipid profile. In healthy subjects, GW501516 significantly lowered fasting plasma triglycerides, ApoB, LDL-cholesterol, and even hepatic fat content [123,124]. Later, similar health benefits were observed in two trials with dyslipidemic patients [125,126]. Nevertheless, the development of GW501516 was discontinued because of its pro-oncogenic properties observed in animal studies [127]. Like GW501516, seladelpar (50 or 100 mg/day) for eight weeks also performed better than placebo or atorvastatin alone in reversing atherogenic dyslipidemia [128,129]. Additionally, seladelpar also improved liver function and was generally well-tolerated [128]. However, larger and longer clinical trials are warranted to yield an in-depth understanding of the clinical efficacy and safety of seladelpar. From these trials, it is clear that different agonists targeting the same PPAR can result in different safety profile and clinical outcomes.

Dyslipidemia often co-manifests with glucose dysregulation, leading to increased risk for prediabetes and T2DM. Thus, several dual PPAR agonists with its dual benefit on glycemic and lipid parameters have also been under clinical development as an anti-dyslipidemia drug. Muraglitazar was the first dual PPARα/γ agonist to be investigated for its lipid-lowering effect in Phase II and III clinical trials (NCT00245388), but the results were not published. In contrast, the safety and efficacy of saroglitazar, a dual PPARα/γ agonist with predominant PPARα activity, in diabetic dyslipidemia have an optimistic outlook. Two Phase III trials showed that saroglitazar could maintain lipid and glucose homeostasis without common side effects of fibrates and TZDs [130,131]. The drug was granted marketing authorization in India in 2013 for diabetic dyslipidemia not controlled with statins [132]. Thus far, no major serious adverse events have been reported; however, long-term cardiovascular safety has not been established [133]. Future clinical trials of saroglitazar will further establish its place in the management of diabetes, dyslipidemia, and associated cardiovascular risk.

The lipid-modifying effect of elafibranor, which is a dual PPAR α/δ agonist, has been confirmed in patients with mixed dyslipidemia [73]. Upon oral administration of elafibranor for four weeks, a significant reduction in fasting plasma triglycerides (−16.7%), γ glutamyl transferase (–19.9%), and LDL-cholesterol (–11%), while HDL-cholesterol was elevated by 7.8% in comparison to placebo [73]. These effects correlated with a reduction in pro-atherogenic apolipoproteins, including ApoB (–14%) and an increase in ApoA2 anti-atherogenic HDL particles (+18%). The promising effects of elafibranor on lipid profiles and various liver enzymes have prompted the developing company (Genfit) to repurpose the drug for the treatment of NASH and PBC (see Section 7).

Genetic disorders cause a small subset of dyslipidemia cases. In this context, the effectiveness of fibrate on various familial dyslipidemia and hypercholesterolemia subtypes differs significantly. For instance, fibrates successfully improved hypertriglyceridemia caused by familial dysbetalipoproteinemia, but not those caused by lipoprotein lipase deficiency or glycerol kinase deficiency [134]. The finding is in line with a crossover study which concluded that a four-week regime with bezafibrate lowered triglycerides and increased HDL-cholesterol in those with familial dysbetalipoproteinemia [135]. Bezafibrate also conferred additional benefits when it was used in combination with statins, suggesting that fibrate/statin therapy could be a better standard-of-care in familial dysbetalipoproteinemia [136]. However, bezafibrate failed to improve the clinical symptoms of patients with X-linked adrenoleukodystrophy [137], CPT2, and very long-chain acyl-CoA dehydrogenase deficiency [138]. Apart from that, seladelpar has also completed a Phase II clinical trial as a therapy for homozygous familial hypercholesterolemia. In the 12-week, single-arm, monthly dose escalation (50, 100, and 200 mg/day) study, eight out of 13 participants had a ≥20% LDL-cholesterol decrease from baseline despite the lack of dose response [139]. In patients with familial combined hyperlipidemia, pioglitazone together with conventional lipid-lowering drugs also significantly improved HDL-cholesterols, myocardial glucose disposal, adiponectin, and ALT besides promoting the fat deposition to subcutaneous adipose tissues [140,141]. However, the present findings are based on small sample sizes owing to the rarity of these genetic disorders. Therefore, larger trials are required to validate the results.

For the past two decades, PPAR agonists have been actively tested for HIV-associated dyslipidemia and lipodystrophy syndrome. The underlying cause for the lipid dysregulation is attributable to highly active antiretroviral therapy (HAART), particularly the use of nucleoside reverse-transcriptase inhibitors, non-nucleoside reverse-transcriptase inhibitors, and protease inhibitors [142]. Multiple studies reported a remarkable decline in plasma triglycerides (>46%) alongside with amelioration of total cholesterol and HDL-cholesterol levels with the administration of fenofibrate in HAART-treated, HIV-positive subjects [143,144,145,146,147,148]. Other fibrates, namely bezafibrate and gemfibrozil, also produced similar favorable outcomes which are comparable to statins [149,150,151]. Fibrates led to more drastic changes in lipid components compared to switching the hyperlipidemia-inducing antiretroviral agents [151]. However, in one study, fibrates failed to modify endothelial function and inflammatory markers in the patients [146]. Gavrila et al. (2005) also demonstrated that fenofibrate did not modulate the blood pressure, glucose, and lipid metabolic parameters, whereas pioglitazone did, over a 12-month regime [152]. Pioglitazone also induced limb fat deposition [153], although one case study suggested that its modulatory effect on subcutaneous fat deposition is limited to non-lipoatrophic regions [154]. In contrast, rosiglitazone had a marginal effect on the lipid profile in HIV-positive patients [155,156,157]. Thus, its use is not recommended [158]. Interestingly, a pilot study pointed out that tetradecylthioacetic acid (pan-PPAR agonist) exerted a notable suppressive effect on total cholesterol, triglycerides, LDL-cholesterol, LDL/HDL cholesterol ratio, and tumor necrosis factor-α [159]. Fibrates, pioglitazone, and tetradecylthioacetic acid appear to modify lipid profile favorably in HIV patients, but their ability to resolve lipoatrophy is not well-characterized.

To recapitulate, PPARα agonists will continue to be the backbone of lipid-lowering drugs, especially with the novel selective PPARα modulator, pemafibrate. The development of seladelpar and dual-PPAR agonists looks wildly exciting considering their optimistic results thus far. The clinical findings of different PPAR agonists in dyslipidemia are summarized in Table 3. Based on the current trend, there will be increasing trials of PPAR agonists on dyslipidemia not only due to lifestyle and metabolism, but also other causes, like genetic disorders, drug-induced, infection, or trauma-related.

## 6. Metabolic Syndrome, Obesity, and Hypertension

### 6.1. Prediabetes and Metabolic Syndrome

Aside from dyslipidemia, PPAR agonists have been widely proposed as a treatment for various premorbid conditions like obesity, glucose intolerance/insulin resistance, MetS, and prediabetes (Table 4). Elafibranor has been tested in several Phase II clinical studies to evaluate its effectiveness in obese patients with prediabetes [72,73]. Glycemic parameters like insulin resistance score, fasting plasma glucose, fructosamine, peripheral, and hepatic insulin sensitivity were significantly improved with elafibranor (80 mg/day) [72,73]. Gene expression analysis of PPARα and δ target genes suggested that elafibranor may be a liver-targeted insulin sensitizer [72].

### 6.2. Obesity

The anti-obesity effect of PPARβ/δ agonists, such as GW501516 and GW677954 (also known as sodelglitazar), has also been examined. GW501516 and GW677954 were developed by GlaxoSmithKline. GW677954 should be considered as a pan-PPAR agonist due to its additional activity at PPARα and γ. In obese men, GW501516 blunted cholesteryl transfer protein activity and modified the biosynthesis of APOC-III, APOA-II, and LpA-I:A-II, leading to lowered VLDL- and LDL-cholesterols, plasma triglycerides, and fatty acid as well as increased HDL-cholesterol [126]. Further development of both drugs was ceased due to carcinogenicity observed in animal testing.

While various PPAR agonists can effectively improve lipid and glycemic aberrations, their use for weight control is not well-supported. In fact, TZDs increase weight gain [11,160]. TZD-induced weight gain is attributable to increased subcutaneous fat depots due to body fat accumulation and redistribution, and body fluid retention [161]. Furthermore, in one clinical study, individuals given rosiglitazone had lower fasting plasma peptide YY and experienced increased hunger [162]. Such an inhibitory effect on peptide YY, which is an appetite suppressor, may also partly explain TZD-induced weight gain. Like TZDs, the weight-lowering effect of PPARα agonist in obese patients is marginal unless coupled with other medications like metformin and orlistat [163,164]. As such, the clinical use of PPAR agonists for weight control is not recommended based on existing evidence.

### 6.3. Hypertension

Elevated blood pressure is one of the most common comorbidities of obesity and metabolic syndrome. One study reported that fenofibrate could significantly lower blood pressure, heart rate, plasma renin activity, and renal vascular resistance in patients with salt-sensitive hypertension, but not salt-resistant hypertension [165]. Likewise, numerous clinical studies have demonstrated a blood pressure-lowering activity of PPARγ agonists in healthy, obese, and diabetic individuals [166,167,168]. In hypertensive patients, pioglitazone triggered the reduction of inflammatory markers, like C-reactive protein (CRP), matrix metalloproteinase-2, and -9, besides improving baroreflex sensitivity and left ventricular diastolic function [169,170,171]. Likewise, favorable changes in endothelial functions, proinflammatory, and prothrombotic biomarkers are also associated with the use of rosiglitazone [172,173]. Nonetheless, the underlying mechanism of the anti-hypertensive and vascular protective effects of PPARγ agonists remain unclear. It is postulated that PPARγ agonists may inhibit the renin-angiotensin-aldosterone pathway to lower sodium and water reuptake, but the hypothesis is in contradiction to its edematous effect [174]. Another study concluded that PPARγ in vascular endothelium plays a key role in attenuating vasoconstriction [175]. These findings underpin a combined modulatory effect on renal and vascular function by PPARγ agonists to account for their anti-hypertensive action. While the blood pressure-lowering effect of TZDs and fibrates is modest at best, such activity may confer additional cardiovascular benefits to individuals with insulin resistance and T2DM.

## 7. Liver Diseases

### 7.1. Non-Alcoholic Fatty Liver Disease (NAFLD)

NAFLD is a spectrum of diseases ranging from the alcohol-independent accumulation of fats in the liver (hepatosteatosis) to inflammation (steatohepatitis), liver fibrosis, and cirrhosis [176]. Due to the lack of approved treatment, existing standard-of-care for NAFLD relies primarily on lifestyle modifications. In this context, PPARs, which are vital lipid regulators and promote anti-inflammatory responses, are ideal targets for NAFLD therapy. The importance of PPARα in NAFLD pathogenesis is demonstrated by a strong negative correlation between PPARα expression in the liver with NASH severity [177]. However, clinical success with PPARα agonists is limited. Fenofibrate and gemfibrozil reliably improve liver function, lipid profile, and insulin sensitivity, but have minimal effects on the histopathology in NAFLD [178,179,180]. Moreover, side effects like impaired kidney function and reversible elevation of serum creatinine and homocysteine are associated with fibrate treatment [181,182]. The occurrence of an adverse incident increases when fibrates are used in combination with certain drugs, particularly gembrozil with cerivastatin, which shows a high incidence rate of rhabdomyolysis [183]. While interest in using fibrates as PPARα agonist for NAFLD and NASH has dwindled, there is much enthusiasm in selective PPAR alpha modulator (SPPARMα) such as pemafibrate to enhance clinical efficacy and minimize side effects. An RCT for the use of pemafibrate in NAFLD patients is underway in Japan, and the results will only be available in 2020 (NCT03350165). Given the superior efficacy and safety profile of pemafibrate for dyslipidemia patients when compared to other fibrates (see Section 5), the outcomes of the ongoing NAFLD trial is highly anticipated.

PPARγ may seem like an unlikely target for NAFLD treatment due to their adipocyte-centric expression and functionality. Ironically, pioglitazone is the only pharmacological therapy recognized by the American Association for the Study of Liver Diseases (AASLD) for use in patients with biopsy-proven NASH, irrespective of their diabetic status [184]. Despite no improvement in fibrosis score, the ameliorative effects of pioglitazone on ectopic hepatic lipid deposition, inflammation, histopathology, and liver function were supported by several RCTs [185,186,187,188,189]. Nonetheless, pioglitazone may be a useful drug candidate for early NASH, despite its limited anti-fibrotic activity.

The initial clinical findings of troglitazone, another PPARγ agonist, also showed improved liver enzymes with a marginal improvement in histological scoring [190]. However, troglitazone was withdrawn from the market after it was found to cause hepatitis. The clinical evidence of rosiglitazone for NAFLD suggests only a temporary benefit from rosiglitazone treatment. A pilot study showed that a 48-week rosiglitazone regime significantly reduced hepatocellular ballooning and hepatic necroinflammation but not liver fibrosis in NASH patients [191]. Likewise, a Fatty Liver Improvement with Rosiglitazone Therapy (FLIRT) study found that rosiglitazone treatment for one year could improve hepatic steatosis and insulin sensitivity, but not liver inflammation and fibrosis [192]. Notably, a two-year extension of the study (FLIRT 2) concluded that the benefits of rosiglitazone in NASH were lost [193].

The combined therapy of rosiglitazone and metformin, supplementing exercise and diet modification, could also attenuate NASH progression [194]. Nevertheless, a similar study pointed out that exercise and diet modification had a better treatment response than either rosiglitazone or metformin alone in NASH [195], highlighting the predominant role of lifestyle modification in NASH treatment. Thus, the short-term use of rosiglitazone offers modest benefits for NAFLD patients, but its long-term efficacy is negligible. The diverse efficacy of different PPARγ agonists in NAFLD and NASH reinforces that notion that the activation of the same PPAR subtype by different agonists does not necessarily lead to similar outcomes.

Given the promising efficacy of certain fibrates and TZDs, the concurrent activation of different PPARs becomes an emerging focus in NAFLD therapy. In dyslipidemic patients, dual- and pan-PPAR agonists can lower liver enzymes and hepatic fat content [72,132]. The positive outcomes lead to several NAFLD trials to explore the clinical feasibility of dual- and pan-PPAR agonists. An example is GOLDEN-505, a Phase II trial that investigated the effects of different elafibranor (dual PPARα/δ agonist) dosages in NASH patients without cirrhosis. A higher proportion of patients on elafibranor (120 mg/day) had lower NAFLD activity score and resolved NASH without fibrosis worsening in addition to the reduction of lipids, glucose, liver enzymes, and inflammatory markers [196]. Elafibranor was well-tolerated, although a slight, reversible increase in serum creatinine was noted [196]. Currently, an ongoing Phase 3 trial (RESOLVE-IT) aims to compare elafibranor to placebo in more than 2000 NASH patients (NCT02704403). The trial is expected to shed more light on the efficacy and long-term safety of the drug.

Saroglitazar, the first approved dual-PPAR α/γ agonist (only in India), has also been trialed as a potential drug for NAFLD because of its beneficial effect on fatty liver in diabetic patients with NAFLD [132]. The outcomes of a Phase III trial (GLAZED) comparing saroglitazar to pioglitazone in NAFLD patients is unpublished (NCT02265276). Meanwhile, saroglitazar is also under active clinical investigation for uncomplicated NAFLD and NASH (NCT03061721; NCT03863574) and those that complicated by other medical conditions, like PCOS (NCT03617263) and liver transplantation (NCT03639623). As the first of its kind, and with positive results from dyslipidemia trials, saroglitazar is a new promising drug for the treatment of NAFLD and NASH.

Another promising dual-PPAR α/γ agonist for NAFLD is lobeglitazone, which has demonstrated a positive therapeutic effect in T2DM patients (see Section 3). A Phase IV trial reported that lobeglitazone significantly reduced intrahepatic fat content, lipid profile, and liver enzymes in diabetic patients with NAFLD [197]. However, it should be noted that the change in hepatic fat content was determined with Fibroscan^®^ instead of liver histology. Therefore, further RCTs using liver histology as the endpoint are vital to validate the real efficacy of lobeglitazone in NAFLD.

According to preclinical evidence, lanifibranor (also known as IVA337) which is a pan-PPAR agonist, can potentially be the right candidate for NAFLD [198]. In preclinical model of cirrhosis, lanifibranor improves portal hypertension and hepatic fibrosis (to be presented at The Liver Meeting^®^ 2019 in Boston, Massachusetts, USA, November 8–12, 2019). However, its clinical development in NAFLD is lagging elafibranor and saroglitazar. Thus far, there are two ongoing Phase II trials which assess the safety, efficacy, and mechanism of lanifibranor in NAFLD and NASH (NCT03008070; NCT03459079). Nonetheless, if the unique pan-PPAR agonistic activity of lanifibranor can translate into exceptional efficacy with minimal safety concern, the drug is likely to benefit not only patients with NAFLD but also many other chronic metabolic diseases. In summary, the resolution of NASH and NAFLD remains an onerous task. Despite numerous preclinical tests suggesting potential in targeting PPARs, human trials are often disappointing. Currently, there are no approved diabetic therapies expect pioglitazone for NASH, but it has drawbacks. There are also many pharmacological differences between PPAR agonists which could affect its efficacy, thus making it hard to conclude which PPAR is the best for improvement in NASH. However, it seems that the next-generation dual-PPAR or pan-PPAR agonists are presently the most promising way to go about tackling this tall task.

### 7.2. Primary Biliary Cholangitis

PBC is an autoimmune liver disease characterized by the presence of anti-mitochondrial antibodies against pyruvate dehydrogenase complex and a unique bile duct pathology [199]. Patients diagnosed with PBC typically develop extensive liver fibrosis and cirrhosis over a few years, while 15% of the patients suffer from liver failure after five years [200]. Currently, ursodeoxycholic acid (UDCA) is the only drug approved for PBC, but about a third of patients are non-responders [201]. Various PPAR ligands have thus been trialed as a new therapeutic approach to supplement UDCA. Co-administration of bezafibrate and UDCA to patients who had an incomplete response to UDCA treatment alone, significantly improved hepatic function and reduced liver fibrosis [202,203,204]. A retrospective cohort study correlated bezafibrate with UDCA combined therapy with reduced risk for liver transplantation and liver-related death [205]. Thus, the combined therapy may improve long-term prognosis of PBC patients. Likewise, the clinical outcomes from another PPARα agonist, fenofibrate was also optimistic as fenofibrate with UDCA could also improve liver biochemistries, leading to a higher complete response rate [206,207,208,209,210]. Taken together, fibrates as adjuvant therapy, aids liver function recovery in PBC patients, particularly among UDCA non-responders. More prolonged and more extensive trials, preferably with histopathological investigation, are warranted to validate the long-term therapeutic effects. Currently, two clinical trials that examine the impacts of bezafibrate in disease progression, quality of life, and cholestatic pruritus intensity are ongoing (NCT02937012; NCT02701166).

In addition to PPARα agonists, other PPAR modulators are also actively subjected to phase II or III clinical testing as a PBC adjunctive therapy. These drug candidates include seladelpar (NCT03602560; NCT03301506; NCT02955602), elafibranor (NCT03124108), and saroglitazar (NCT03112681). It is worth mentioning that elafibranor was granted Breakthrough Therapy Designation by the U.S. FDA for the treatment of PBC. On the other hand, a Phase II trial featuring seladelpar was terminated early due to overly high elevation of ALT, likely attributable to high seladelpar dosages (50 and 200 mg/day) [211]. Resultantly, three subsequent clinical trials, as mentioned above use lower dosages (2–10 mg/day) to minimize untoward events. Considering the clinical success of PPARα agonists in PBC therapy, the efficacy of other PPAR agonists are also awaited with great expectations. Hence, the outcomes from ongoing trials may help to formulate better therapeutic approaches to treat PBC in the future.

### 7.3. Hepatitis C

Hepatitis C is a form of viral hepatitis caused by the Hepatitis C Virus (HCV) that can increase the risk of cirrhosis and liver cancer. In addition to liver injury, hepatic steatosis and insulin resistance are common manifestations which may aid viral replication and survival [34]. Therefore, PPARγ agonists have been proposed to alleviate these symptoms and delay hepatitis C deterioration. Yet, one-year treatment with farglitazar (PPARγ) failed to lessen stellate cell activation and fibrosis in chronic hepatitis C patients who did not respond to anti-viral therapy (pegylated interferon alpha/ribavirin) [212]. In a separate trial, pioglitazone also did not confer any benefit to non-responders [213]. Unlike those who are resistant to anti-viral therapy, in treatment-naïve hepatitis C patients, pioglitazone not only reduced HCV RNA after a 14-day course [214] but also resulted in a higher rate of sustained viral response when used in combination with anti-viral therapy [215]. Collectively, by acting on steatosis and insulin resistance, pioglitazone may be beneficial in early stage or treatment-naïve hepatitis C patients, but less effective in those who are resistant to anti-viral therapy. Despite the promising results, clinical development of PPARγ agonists for hepatitis C was stopped when new and more potent pharmacotherapy, direct-acting antivirals, was approved by the FDA in 2011 for hepatitis C treatment [216]. The clinical results of different PPAR agonists in NAFLD, NASH, PBC, and hepatitis C are summarized in Table 5.

## 8. Kidney Diseases

### 8.1. Chronic Kidney Disease (CKD)

CKD is characterized by persistent, low-grade inflammation of the kidneys, which results in a gradual loss of renal function over time [218]. It is also a common renal complication of many chronic diseases like T2DM, hypertension, and MetS. The inflammatory and metabolic components make PPAR agonists potential drug candidates for CKD. Pioglitazone is well-tolerated in patients with CKD [219]. Indeed, pioglitazone improved the visceral-to-subcutaneous fat deposition, adipokine profile, hepatic insulin sensitivity, and circulating CRP in non-diabetic patients on dialysis [220]. Similar improvements in adiponectin and CRP were also found in obese, diabetic, or insulin-resistant patients with end-stage renal failure (NCT01301027). Furthermore, endothelial function, including flow-mediated dilatation, arterial compliance, and pulse-wave velocity, were not affected by pioglitazone (NCT00586261) and rosiglitazone [221] in CKD patients. A post hoc analysis of PROactive trial revealed that pioglitazone could reduce the incidence of all-cause mortality, myocardial infarction, and stroke in diabetic patients with CKD [222]. Ironically, there was a more significant decline in kidney function in pioglitazone cohort [222]. An ongoing Phase IV trial is looking into the role of pioglitazone in sympathetic nervous system to understand the effect of the PPARγ agonist in lowering cardiovascular risk among CKD patients (NCT03471117). Based on existing evidence, the cardiovascular benefits of pioglitazone in CKD looks optimistic, but the underlying mechanism may not be a direct amelioration of vascular and renal impairment. Future studies should address this question by looking into the effects of TZDs on renal-related endpoints like the progression of kidney disease, glomerular filtration rate, and renal function biomarkers. The outcomes will help to capture the subsets of CKD patients who will truly benefit from the treatment.

### 8.2. Other Kidney Diseases

Apart from CKD, PPARγ agonists have also been trialed in other kidney complication. In renal transplant recipients with newly diagnosed impaired glucose tolerance, pioglitazone reliably improved the insulin sensitivity [223]. This finding was confirmed by another trial that showed pioglitazone significantly reduced carotid intima-media thickness, suggesting a reduction of cardiovascular risk in renal allograft recipients [224]. These results suggest a role for pioglitazone in the management of post-renal transplantation complications.

Maalouf et al. (2019) showed that pioglitazone improved some features of MetS, reduced net acid secretion, and increased urine pH in patients with uric acid nephrolithiasis, suggesting lower kidney stone formation [225]. Although such treatment is unlikely to replace alkali administration to prevent kidney stone formation, the results established insulin resistance as an important factor of low urine pH. This provides a basis for the use of PPARγ agonists as a treatment for MetS and preventive approach for kidney stone.

A Phase I pilot study was conducted to examine the safety and efficacy of rosiglitazone in resistant focal segmental glomerulosclerosis (NCT00193648). Pioglitazone was also trialed in a Phase II study for autosomal dominant polycystic kidney disease (NCT02697617). The outcomes of both trials remain unpublished. The preliminary results from the above trials will be a dominant factor for further exploration of pioglitazone in other renal-related diseases. Table 6 summarizes the clinical evidence of different PPAR agonists in kidney diseases.

## 9. Neurodegenerative Diseases and Neurological Dysfunction

### 9.1. Alzheimer’s Disease and Parkinson’s Disease

Expanding interests into metabolic disorders have led to the proposal of how metabolic disorders and Alzheimer’s disease have overlapping risk factors, which generated interests of PPAR in Alzheimer’s disease. In a pilot Phase II clinical trial conducted on patients with mild Alzheimer’s disease and amnestic mild cognitive impairment, a 6-month course of rosiglitazone improved clinical outcomes like better delayed recall, selective attention, and stable plasma level of amyloid β-42 peptide [226]. The positive outcomes suggest that rosiglitazone may be a novel strategy for cognitive decline, subsequently driving GlaxoSmithKline to spearhead further clinical trials by amassing thousands of subjects that were stratified into APOE ε4 carriers or non-carriers for the study of extended release rosiglitazone under subsequent REFLECT program. Results from a Phase II trial showed that extended-release rosiglitazone for a year was well-tolerated and could enhance cerebral glucose metabolism, but not displaying clinical improvement in patients with mild to moderate Alzheimer’s disease [227]. A larger Phase II trial also concluded that rosiglitazone did not improve cognitive function of patients with mild Alzheimer’s disease, although exploratory subgroup analysis revealed that patients without APOE ε4 allele, a strong genetic risk factor of Alzheimer’s disease, might be more responsive with rosiglitazone [228]. However, further investigation of the efficacy of rosiglitazone in Alzheimer’s disease, either as a monotherapy or an adjunctive therapy to acetylcholine esterase inhibitors, in five Phase III clinical trials, did not yield meaningful outcomes in cognition and global function, even when the patients were stratified into APOE ε4 carriers or non-carriers [229,230]. With many trials done, but no important findings of the potential therapeutics of rosiglitazone on Alzheimer’s disease, GlaxoSmithKline has ceased the development of rosiglitazone as Alzheimer’s disease therapy.

In a TOMMORROW trial, pioglitazone was tested as a preventive medication for healthy subjects with a strong predisposition to mild cognitive impairment due to Alzheimer’s disease. The trial was prematurely terminated due to the lack of efficacy of pioglitazone in preventing the onset of cognitive impairment among high-risk patients (NCT01931566). Other clinical trials that aimed to assess the therapeutic effects of pioglitazone in mild cognitive impairment and Alzheimer’s disease also did not identify improvement in cognition, neuropsychiatric symptoms, global function, and daily activities [231,232]. In line with Alzheimer’s disease trials, pioglitazone also failed to modify the progression of early Parkinson’s disease according to a Phase II trial [233]. Thus far, mounting evidence from past trials strongly points out the ineffectiveness of PPARγ agonists in ameliorating neurodegenerative diseases. Hence, further clinical development in these aspects is not recommended unless stronger evidence, along with concrete pharmacological interaction that supports the use of PPARγ agonists arises. Despite the failure of PPARγ agonists, a new Phase I trial piloting a PPARα agonist-gemfibrozil, for pre-dementia Alzheimer′s patients is on-going (NCT02045056). The study explores the impacts of gemfibrozil on Alzheimer’s disease biomarkers like miRNA-107, amyloid β-40, and -42 peptides in serum, and cerebrospinal fluid. Outcomes of the trial will determine the feasibility of repurposing gemfibrozil as well as other PPARα agonist for Alzheimer’s disease therapy.

### 9.2. Amyotrophic Lateral Sclerosis

Amyotrophic lateral sclerosis (ALS) is a lethal neurodegenerative disease resultant of motor neuronal death, with clinical presentation of muscle degeneration, paralysis, respiratory distress, and eventual death from respiratory distress. Recent ALS studies involving SOD1 mutant mouse model were indicative of the protective effects of anti-inflammatory compounds in reducing inflammation-induced neuronal death in ALS [234]. Pioglitazone, with its anti-inflammatory properties, may play a neuroprotective role in alleviating ALS. Ironically, pioglitazone′s side effect of moderate weight gain may improve survival through rescuing energy deficiency for ALS patients. Despite reported successes of pioglitazone in animal ALS models [235,236], these successes were not observed in the clinical trials that piloted pioglitazone in the amelioration of ALS. Dupuis et al. (2012) reported no improvement upon co-administration of 45 mg/day of pioglitazone and 100 mg/day of riluzole when compared to a placebo group, with increased patient death (30:24 by the end of two years) and a hazard ratio of 1.21 at the end of the trial [237]. The trial was prematurely ended on the reason of futility from the adverse events of ALS disease progression, despite well-tolerance of pioglitazone. In another Phase I/II clinical trial, co-administration of riluzole, tretinoin, and pioglitazone did not delay the disease progression of ALS [238]. Existing clinical evidence does not support the use of pioglitazone in ALS treatment. The discrepancy between clinical and preclinical findings may be attributable to the heterogeneity of genetic spectrum of ALS which the SOD1 mutant ALS mice are unable to reproduce.

### 9.3. Multiple Sclerosis

Chronic demyelination in the central nervous system mediated by a targeted autoimmune response and its implications in the disease progression of multiple sclerosis has also been extensively studied via experimental autoimmune encephalomyelitis models [239]. Only limited clinical evidence exists on the potential therapeutics of PPARγ agonists, pioglitazone and CHS-131 (also known as INT-131) for multiple sclerosis. Evidence was based on the inflammatory properties of these agonists in the preclinical model. Pioglitazone or placebo co-administered with interferon β-1α to patients with relapsing-remitting multiple sclerosis showed no clinical improvement or adverse events through expanded disability status scale, despite a promising significant reduction in gray matter atrophy and a positive trend of lower lesion load in subsequent MRI follow-ups [240]. Another PPARγ agonist, CHS-131, was also trialed, with reported lower new contrast-enhanced lesions of 52% and 21% in high (3 mg CHS-131/day) and low (1 mg CHS-131/day) dose cohorts, respectively, when compared with placebo [241]. The relapse rate was also lowered by 33% and 24% in high- and low-dose cohorts relative to the placebo-treated group [241]. These two trials highlight the potential of PPARγ agonists for multiple sclerosis. The need to upscale these trials to test PPARγ agonists′ efficacies and even possible counter-interactions has become increasingly critical, especially when these agonists are more widely applied to patients in the clinical settings for other medical disorders.

### 9.4. Other Neurological Disorders

The efficacy of PPAR agonists was also examined for other neurological disorders like epilepsy (EUCTR 2011-005433-39), post-herpetic neuralgia (NCT01318226), and Friedreich’s ataxia (NCT00811681). Adjunctive therapy with fenofibrate for six months markedly reduced seizure frequency in patients with drug-resistant nocturnal frontal lobe epilepsy [242]. ATx08-001 (also known as FK614), a non-TZD PPARγ agonist has been examined for its safety and analgesic properties in individuals with post-herpetic neuralgia (NCT01318226), but no published information regarding the trial outcomes is available. Due to an improved antioxidant mechanism, pioglitazone has also been proposed and trialed as a therapy for Friedreich’s ataxia, a rare neurological disorder arising from recessive genetic inheritance of a mutated Frataxin (FXN) gene on chromosome 9q13 (NCT00811681). The data remain unpublished to date, one of the investigators did mention that pioglitazone was unable to improve neurological function in patients with Friedreich’s ataxia [243]. While PPAR agonists have been tested in many neurological disorders, the outcomes of most trials are mostly negative or unavailable (Table 7). Hence, future investigation in this aspect is not encouraged unless stronger evidence arises.

## 10. Psychiatric Disorders

### 10.1. Addiction/Substance Dependency

Emerging preclinical studies suggested that PPARs can potentially be a target for curbing addiction and substance abuse such as alcoholism as well as drug/cigarette (cocaine, nicotine, and opioid) dependence [244]. These findings spurred pilot clinical trials to determine PPAR agonists in reducing dependencies on these substances. A Phase II clinical trial investigated the effectiveness of fenofibrate on alcoholism (NCT02158273). Measurements on an intention to drink, loss of control, relief craving, urge intensity, and number of drinks after one week associated a minor improvement with fenofibrate, but two other trials that explored the clinical value of gemfibrozil and pioglitazone respectively in alcoholism were terminated (NCT03539432; NCT01631630). Gemfibrozil and pioglitazone were also trialed in several independent clinical trials for nicotine dependence/smoking. Gendy et al. (2018) employed a crossover experimental design (two-week treatment phases separated by one-week washout period) and reported no significant difference between gemfibrozil- and placebo-treated smokers in number of days of abstinence, willingness to opt for de-nicotinized cigarettes, as well as physiological changes and self-reported craving upon exposure to a smoking cue (cigarette) [245]. These findings were in line with that of fenofibrate [246]. Another recent trial on gemfibrozil, however, revealed that gemfibrozil for nine weeks decreased mean exhaled carbon monoxide, but increased the Heaviness of Smoking Index, which is an indicator of the nicotine dependency severity (NCT02638597). Likewise, pioglitazone was found to reduce nicotine craving in heavy smokers [247]. The ameliorative effect of pioglitazone on craving intensity has also been reported in a Phase I/II clinical trial on cocaine abuse [248]. In the study, not only was pioglitazone associated with a significant reduction in self-report craving assessments, but also a notable improvement in white matter integrity [248]. Contradictorily, trials on opioid dependency did not identify any beneficial outcomes with pioglitazone in drug abuse tendency and withdrawal symptoms [249,250]. In general, all the trials above had small sample sizes, rendering the efficacy of PPARα or PPARγ agonists in smoking cessation and substance abuse inconclusive. Reconsiderations for further trials of PPARα and PPARγ agonists can be made in the perspectives of the somewhat positive results from specific trials.

### 10.2. Major Depressive Disorder and Bipolar Depression

PPARγ agonists to address mood disorders, especially major depressive disorder and depressive episodes in bipolar disorder, have been tested in a few clinical trials. In a Phase IV trial, an eight-week adjunctive treatment with pioglitazone drastically improved depression symptoms, social functionality, self-reported depression severity, and clinician-rated anxiety severity of patients with bipolar depression [251]. Similar benefits were also obtained with rosiglitazone as an adjunct therapy in bipolar depression, suggesting that PPARγ could potentially modulate mood and emotion [252]. In major depressive disorder, 12-week pioglitazone monotherapy reduced clinician-rated depressive symptoms (NCT00671515). Similarly, combined therapy of pioglitazone and citalopram, which is an antidepressant, also led to a better response, higher remission rate, and frequency of early improvement (reduction of depression scoring within the first two weeks of treatment) compared to citalopram alone [253]. Another trial reported that the anti-depressive effect of pioglitazone was only found in patients with comorbid insulin resistance and appeared more potent in younger patients, implying a linkage between depression and metabolic dysregulation [254]. Indeed, in schizophrenic patients, pioglitazone also significantly improved depressive symptoms and antipsychotics-induced metabolic dysfunction but failed to modify cognitive performance although a regional discrepancy (American and Chinese subjects) in the results was observed [255]. It is speculated that the improvements in depressive symptoms are partly mediated by the anti-inflammatory and metabolic regulatory effect of PPARγ, as evidenced by the correlation between depressive symptomatology scoring with a pro-inflammatory cytokine, IL-6 [251], and an adipokine, leptin [256], when pioglitazone was prescribed to patients with bipolar depression. Meanwhile, two trials which investigate the therapeutic effects of pioglitazone (EUCTR 2014-003803-31-ES) and bezafibrate (NCT02481245) respectively, in bipolar depression are underway. Existing clinical studies that explore the effects of PPARγ agonists on major depressive disorder and bipolar depression mainly suffer from small sample size. Nonetheless, most of them seem to agree on the potential anti-depressive activity of pioglitazone and rosiglitazone, highlighting the pertinence of further investigation to validate the clinical efficacy.

### 10.3. Autism Spectrum Disorder

In the aspect of autism spectrum disorder, the clinical feasibility of pioglitazone was initially assessed in 25 autistic children in an open-label trial. This results highlighted a significant reduction in irritability, lethargy, stereotypy, and hyperactivity with pioglitazone [257]. Later, the findings of a RCT with pioglitazone as an adjunctive treatment to risperidone also mirrored that of the previous study [258]. Inadequacy in the mode of assessments and dosage safety missing in the earlier studies led to a Phase II clinical trial which concluded that in autistic children, pioglitazone is well-tolerated at 0.75 mg/kg/day [259]. Pioglitazone also favorably modifies behavioral symptoms of autistic children, along with a significant reduction of pro-inflammatory IL-6 and increment of anti-inflammatory IL-10 [259]. These trials are indicative of the benefits pioglitazone can confer to the autism spectrum disorder patients, with similar conclusions of upscaling pioglitazone as a mono- or adjuvant therapy in subsequent trials, if any. The clinical outcomes of different PPAR agonists in psychiatric disorders are summarized in Table 8.

## 11. Autoimmune Diseases

### 11.1. Rheumatoid Arthritis

The use of TZDs can be extended to treat several autoimmune diseases. In rheumatoid arthritis, drug efficacy in clinical settings was mostly measured by the Disease Activity Score in 28 joints (DAS28) based on CRP level and erythrocyte sedimentation rate (ESR), both of which are inflammatory markers commonly elevated in rheumatoid arthritis, as well as parameters such as Global Health, swollen joint count, and tender joint count. In two randomized, crossover studies, the addition of 45 mg/day pioglitazone to rheumatoid arthritis patients’ baseline disease-modifying antirheumatic drug (DMARD) therapy yielded a significant improvement in patient-reported global health and pain score, along with a significant reduction in the DAS28-CRP level [260,261]. Adjunctive pioglitazone therapy, however, showed no significant changes in DAS28-ESR, swollen joint count, and tender joint count in comparison to the placebo-treated arm [260]. Similarly, another study which used a combination of pioglitazone with methotrexate also reported a significant reduction of DAS28 score and CRP, without significant differences in the swollen joint and tender joint counts [262]. These results suggest that pioglitazone can be a potential adjuvant therapy used in conjunction with other drugs to treat RA.

### 11.2. Systemic Lupus Erythematosus

Pioglitazone was also examined for the treatment of SLE, an autoimmune disease which is highly predisposed to cardiovascular risk [263]. A double-blind RCT with pre-menopausal women with SLE reported a significant reduction of 70.9% in CRP levels and 34.9% in serum amyloid A upon a 12-week course with 30 mg/day pioglitazone [264]. Such a marked reduction in inflammation occurred in parallel to other benefits like enhanced insulin sensitivity and improved cholesterol profile, suggesting a potential role of pioglitazone in the treatment of SLE with cardiovascular risk [264]. Currently, there is another ongoing trial examining the efficacy of pioglitazone on vascular function, inflammatory response, and lupus disease activity in SLE patients (NCT02338999). Clearly, the finding from this ongoing trial is highly anticipated as a positive outcome will fuel additional experimental investigation and future trials of pioglitazone in SLE.

### 11.3. Other Autoimmune Diseases

Pioglitazone exhibited a significant reduction in the severity of pulmonary alveolar proteinosis in Csf2 deficient mice, along with an increased ability of cultured macrophages to clear surfactant in vitro. These results prompted an ongoing Phase I/II trial on the treatment of autoimmune pulmonary alveolar proteinosis to stimulate the ability of alveolar macrophages to clear surfactant (NCT03231033), which can provide more insight about the immune-modulatory effect of pioglitazone. On the other hand, lanifibranor (pan-PPAR agonist) has been examined in a Phase II trial for diffuse cutaneous systemic sclerosis, an autoimmune disease of the connective tissues and has extensive complications in skin, kidneys, heart, lungs, and gastrointestinal tract (NCT02503644). Although the detailed results were unpublished, the developing company (Inventiva) announced that lanifibranor failed to improve skin thickness and disease progression. Further development of lanifibranor in diffuse cutaneous systemic sclerosis is discontinued. In short, the clinical results of pioglitazone in autoimmune diseases look promising, whereas the efficacy of other PPAR agonists is rarely investigated. Existing clinical evidence of PPAR agonists in autoimmune diseases are tabulated in Table 9.

## 12. Inflammatory and Infectious Diseases

### 12.1. Malaria

Several clinical studies also evaluated the anti-inflammatory effects of TZDs against various inflammatory and infectious diseases. In a Phase I/II study, patients with uncomplicated *Plasmodium falciparum* malaria receiving rosiglitazone in addition to standard anti-malaria regimens had significantly faster 50% and 90% parasite clearance times as well as a significant reduction in inflammatory responses to infection. However, serum AST or ALT levels did not improve in comparison to placebo-treated patients [265]. In contrast, adjunctive treatment with rosiglitazone in another Phase II trial was not superior to placebo in improving AST, ALT, hematocrit, hemoglobin, mean parasite density, and the median time to parasite clearance, despite its safety and well-tolerance [266]. These studies suggest that rosiglitazone is well-tolerated among malaria patients. Combining rosiglitazone with anti-malaria therapy demonstrated a certain extent of clinical efficacy to attenuate inflammatory response, but the modest effectiveness is unlikely to be translated into clinical use.

### 12.2. Ulcerative Colitis

The clinical efficacy of TZDs was also evaluated in ulcerative colitis, as measured by improvement in the quality of life and disease activity. Patients with mild to moderately active ulcerative colitis showed higher rates of clinical response and remission, and improvement in their quality of life when given rosiglitazone for 12 weeks [267]. Similarly, excellent tolerability and a significant reduction in disease activity were observed when patients with active distal ulcerative colitis were treated with rosiglitazone enema treatment [268]. Indeed, the expression of PPARγ was tremendously suppressed in the inflamed intestinal mucosa, whereas TZD-dependent PPARγ activation could exert a local anti-inflammatory effect in the gut to treat ulcerative colitis [268]. The positive results from clinical studies do support the repurposing of rosiglitazone to treat ulcerative colitis.

### 12.3. Asthma

PPARγ involvement in asthma exacerbation has been observed [269]. Rosiglitazone administered at 4 mg twice daily did not improve the mean FEV1 in asthma patients challenged with allergen inhalation, but was associated with a 15% reduction in the weighted mean late asthmatic reaction and decreased inflammatory markers [270]. In contrast, pioglitazone did not show significant changes in the lung function, asthma control, airway inflammation, and quality of life in asthma patients [271,272,273]. In fact, the use of pioglitazone precipitated notable adverse events like increased use of short-acting β2-agonists (SABA), fluid retention, and weight gain, rendering the three studies terminated prematurely [271,272,273]. As such, the lack of efficacy and significant safety concerns exclude the application of TZD for asthma treatment. Nonetheless, these studies highlighted the fact that different TZDs can display vastly different safety profiles.

### 12.4. Psoriasis

Oral administration of TZD has been reported to improve psoriasis, an inflammatory skin disorder. Based on a meta-analysis, pioglitazone was associated with a significant reduction of Psoriasis Area and Severity Index compared to placebo, but such an improvement was not observed in the rosiglitazone-treated cohort [274]. The combined therapy of pioglitazone with acitretin, a retinoid, also led to better improvement in PASI among moderate to severe plaque psoriasis, relative to acitretin therapy only [275]. Although the beneficial effect of pioglitazone is indicated, it should be noted that the conclusion was established based on limited studies, thus highlighting the necessity of further investigation.

### 12.5. Endometriosis

In three case studies of women with endometriosis, 4 mg/day rosiglitazone showed improvements in the severity of symptoms and pain levels with a reduction in pain medication in two of these patients, with one showing no change [276]. A follow-up trial that used pioglitazone to relieve pain and reduce cytokine levels in endometriosis was prematurely halted (NCT01184144). Evidence for the use of TZDs in endometriosis is limited and inconclusive.

### 12.6. Cystic Fibrosis

The efficacy of pioglitazone was evaluated in the treatment of cystic fibrosis. A 28-day Phase I clinical trial of 30 mg/day pioglitazone did not show any improvement in all of the tested sputum inflammatory mediators in healthy individuals. This finding was believed to be due to the inadequate dose of pioglitazone, short duration of the study, and small sample size [277]. Two single-arm trials that recruited cystic fibrosis patients and examined the effects of pioglitazone on sputum inflammatory markers and airway inflammation have been completed (NCT00719381, NCT00322868). Even though the findings are not published, the results provided in the registered clinical trial database (NCT00322868) showed that one-month treatment with pioglitazone did not modify most of the inflammatory markers, including white cell count, neutrophil count, active elastase, TNFα, IL-1β, IL-6, and IL-8 in sputum specimens of treated patients. Based on existing evidence, the use of PPARγ agonists to modulate inflammation in cystic fibrosis is not supported.

### 12.7. Other Inflammatory and Infectious Diseases

Other inflammatory diseases that underwent clinical trials with TZDs include sepsis where pioglitazone showed a significant reduction in inflammatory markers: IL-6, IL-8, resistin, and TNF-α in patients with severe sepsis or septic shock [278], lung inflammation where Chen et al. (2018) concluded that pioglitazone had no anti-inflammatory effect in healthy volunteers based on ^18^F-FDG PET/CT imaging [279], and chronic granulomatous disease where pioglitazone reduced CRP from 24.4 to 13.1 mg/L in a five-month old patient [280]. The efficacy of pioglitazone to treat patients with chronic granulomatous disease (NCT03080480), gastric phlogosis due to *Helicobacter pylori* infection (EUCTR 2005-001218-42), as well as lung inflammation in alcoholic individuals (NCT03060772) is being assessed in ongoing trials. On the other hand, in FFAME trial, fenofibrate failed to attenuate lipopolysaccharide-induced inflammation in healthy individuals [281].

Taken together, PPARγ agonists seem to have some implications in the treatment of certain infectious or inflammatory diseases, including malaria, ulcerative colitis, psoriasis, and septic shock (Table 10). While the successful clinical translation of PPARγ agonists to treat these diseases remains questionable, the anti-inflammatory activity demonstrated is likely to encourage more trials of TZDs in this aspect. Unlike PPARγ agonists, clinical evidence of the anti-inflammatory effect of other PPAR modulators is scarce.

## 13. Malignancy

### 13.1. Head and Neck Squamous Cell Carcinoma (HNSCC)

The aggressive nature of head and neck squamous cell carcinoma (HNSCC) demands better therapeutic options beyond current treatment paradigms. As candidates in the fervent search, pioglitazone and rosiglitazone have been incorporated for the prevention of HNSCC by targeting leukoplakia, which is a precancerous lesion that may develop into squamous cell carcinoma. Currently, two trials have been completed with regards to pioglitazone as monotherapy for HNSCC. In a Phase II trial, 71% of the subjects with either hyperplastic, dysplastic, or oropharyngeal leukoplakia had a partial response to pioglitazone therapy as characterized by disappearance or reduction of lesions, dysplasia, or hyperplasia [282]. A follow-up trial then demonstrated a superior effect of pioglitazone to placebo (46% vs. 32%) despite being terminated early due to slow accrual of patients (NCT00951379). The effectiveness of rosiglitazone on oral leukoplakia was also tested in another study (NCT00369174), but the findings were not released. Additionally, a Phase II clinical trial (NCT02917629) testing the efficacy of pioglitazone on stage I–IV oral cavity or oropharynx cancer is currently actively recruiting. Despite promising results from the completed trials, the scale in these trials may be deemed too small to conclude on the potential of pioglitazone for HNSCC. Larger trials are necessary to draw a conclusion about the clinical advantages of pioglitazone and rosiglitazone in oral leukoplakia and HNSCC.

### 13.2. Thyroid Cancer

Efatutazone (also known as CS-7017 and RS5444), a PPARγ agonist developed by Daiichi Sankyo, has been clinically assessed in anaplastic thyroid carcinoma. The results of a Phase I/II study showed that the adjunct therapy of efatutazone to paclitaxel significantly slowed down disease progression and survival duration in a dose-dependent manner [283]. This promising results led to an ongoing a Phase II study that further investigates the impacts of efatutazone in anaplastic thyroid carcinoma progression (NCT02152137). A small portion of thyroid carcinomas carries an oncogenic paired box 8 (PAX8) and PPARγ fusion protein. In a thyroid cancer patient with such mutation, pioglitazone for 6 months shrank an acetabulum metastatic lesion (6 to 3.9 cm) and lowered thyroglobulin by 97% [284]. The benefits were observed even after pioglitazone treatment has ceased for 13 months. While a meaningful conclusion about the efficacy of pioglitazone cannot be drawn from a single case study, the positive result does support follow-up investigations of the PPARγ agonist in this subset of thyroid cancer patients. Rosiglitazone was also assessed for patients with thyroglobulin-positive and radioiodine-negative differentiated thyroid cancer. Five out of 20 subjects given rosiglitazone had a partial treatment response as exemplified by the increased radioiodine uptake while the rest had stable or continual disease progression [285]. No subject had a complete or partial response to rosiglitazone at three month follow-up [285]. It was, therefore, concluded that rosiglitazone is unable to halt the progression of differentiated thyroid cancer.

### 13.3. Lung Cancer

Efatutazone was also tested on non-small cell lung carcinoma (NSCLC) as adjuvant therapy to antineoplastic agents. A Phase I trial showed that a combined therapy of efatutazone, paclitaxel, and carboplatin induced partial responses in 37.5% (six out of 16) subjects with either metastatic or unresectable NSCLC [286]. A Phase II trial that examined the safety and effectiveness of the combined therapy on progression-free survival in metastatic NSCLC has been completed, but no data has been released (NCT00806286). Likewise, a combined therapy of efatutazone and erlotinib conferred partial responses in 36% (five out of 14) of the subjects with NSCLC [287]. This was also followed up with a Phase II trial (NCT01101334). Clinical development of efatutazone for NSCLC has been terminated, and the result of the trial was not published. Other PPARγ agonists were also identified for subsequent trials. Pioglitazone was trialed both as a preventive measure and a curative agent. Wigle et al. [288] and Keith et al. [289] examined pioglitazone as a chemoprevention mean for lung carcinoma among high-risk smokers or early-stage NSCLC patients. The results revealed reduced cancer cell proliferation with pioglitazone treatment. While the efficacy of PPARγ agonists for NSCLC remains inconclusive, another ongoing study (NCT02852083) may provide an additional perspective for pioglitazone in addressing NSCLC.

### 13.4. Colorectal Cancer

Efatutazone showed some promising results in two clinical trials for colorectal cancer. Subjects who responded to first-line chemotherapy had longer progression-free survival and a higher overall survival rate (NCT00986440). Adjunct therapy of efatutazone to the FOLFIRI chemotherapy regimen (NCT00967616) only achieved a modest improvement in progression-free survival duration [290]. The developing company Daiichi Sankyo has discontinued any further trials on efatutazone against colorectal cancer.

### 13.5. Prostate Cancer

PPARγ agonists as a monotherapy for maintenance therapy was studied during prostate cancer remission. Rosiglitazone did not delay disease progression [291]. A Phase II clinical study in patients with advanced prostate cancer using troglitazone revealed an unexpected prolong stabilization of prostate-specific antigens [292]. This observation suggests that PPARγ may serve as a biological modifier in human prostate cancer. A combined therapy consisting of pioglitazone, imatinib, etoricoxib, dexamethasone, and low-dose treosulfan were evaluated in patients with castration-resistant prostate cancer and found that 23 out of 61 subjects responded to the regimen with mean prostate-specific antigen decreasing by 97% [293]. Another clinical trial also tested a combined therapy with PPARγ agonist (rosiglitazone/pioglitazone), fenofibrate, and calcitriol in castration-resistant prostate cancer, but was terminated early due to low accrual (EUCTR 2006-001398-44). TZDs may be beneficial to a specific subtype of prostate cancer, and their therapeutic potential in this disease should be further investigated.

### 13.6. Blood Cancer (Leukemia, Lymphoma, Myeloma)

The toxicity and ineffectiveness of many chemotherapeutic drugs require the continued search for better and safer therapeutic drugs. Numerous preclinical trials have highlighted the anticancer potential of many PPAR agonists. A small clinical study reported that the combinational therapy of bezafibrate and medroxyprogesterone acetate has minimal hematological toxicity and a decent treatment effect in acute myeloid leukemia (AML) patients who were not suitable for intensive chemotherapy [294]. Interestingly, the same benefits were no longer observed with the same regime at higher dosage [295]. Pioglitazone is being assessed for AML as a combined therapy with low-dose azacitidine and all-*trans*-retinoic acid (NCT02942758).

Current trials of PPARγ agonists on chronic myeloid leukemia (CML) often act as a curative add-on to tyrosine kinase inhibitors, which are effective in refractory CML. A few trials have been initiated to study pioglitazone with various tyrosine kinase inhibitors, but their findings remain unknown. The majority of the trials are either at an early stage of recruitment (NCT02767063, NCT02889003), ongoing (NCT02852486), terminated (NCT02730195), or of unknown status (NCT02687425). In a completed Phase II trial, pioglitazone with imatinib malate resulted in long-term suppression of BCR-ABL1 expression in most of the CML patients [296].

A 16-week open label study on cutaneous T-cell lymphoma, using combinational therapy of bexarotene and rosiglitazone in four subjects, showed a reduction in skin scores of scaling in two patients and amelioration of pruritus in three of four subjects [297]. Another clinical trial assessed the feasibility of bezafibrate with medroxyprogesterone acetate as therapeutics for endemic Burkitt’s lymphoma, a disease which is associated with chronic malaria. The disease progression and clinical response followed a dose-dependent trend, indicating good efficacy of the combined therapy [298]. The positive results should be validated in larger RCTs to determine its benefits in endemic Burkitt’s lymphoma.

Several PPAR agonists have also been trialed in multiple myeloma patients, including fenofibrate (NCT01965834), efatutazone (NCT01504490), and pioglitazone (EUCTR 2008-002768-32; NCT0101243). The first two of which were terminated early for unclear reasons whereas those that involve pioglitazone are ongoing.

### 13.7. Skin Cancer

Tumor promoting inflammation is a hallmark of cancer. Controlling such inflammatory processes may aid in prolonging progression-free survival in patients with metastatic melanoma. Hart et al. (2016) combined pioglitazone, etoricoxib, low-dose trofosfamide, and temsirolimus for stage IV melanoma, and concluded that the combined therapy could potentially prolong progression-free survival duration [299]. Independently, pioglitazone with rofecoxib in conjunction with low-dose chemotherapy in metastatic melanoma also reached a similar conclusion [300], suggesting that multi-modal therapy that includes PPARγ agonists benefits melanoma patients. Currently, a Phase II clinical trial testing the efficacy of pioglitazone as a monotherapy for skin squamous cell carcinoma is recruiting participants (NCT02347813). 

### 13.8. Liposarcoma

PPARγ agonists have been considered as agents for differentiation therapy in liposarcoma [301]. Thus far, mixed clinical results have been reported. In a small pilot study on liposarcoma, troglitazone markedly increased lipid accumulation in the tumor biopsies besides promoting adipocyte differentiation and attenuating tumor cell proliferation [302]. Troglitazone has completed a Phase II study on advanced or metastatic liposarcoma (NCT00003058), but no result is available. In contrast, rosiglitazone did not induce notable histological and clinical response in liposarcoma patients [303]. Another Phase II trial on the effectiveness of rosiglitazone in liposarcoma has been conducted, but no data was released. Overall, sparse findings support the use of TZDs as differentiation therapy in liposarcoma.

### 13.9. Breast Cancer

In refractory breast cancer, troglitazone failed to improve disease progression [304]. A lack of effect was observed in breast cancer patients given short-term rosiglitazone treatment (2–6 weeks). Based on these results, PPARγ agonists have little clinical value in breast cancer therapy [305].

### 13.10. Brain Cancer

Currently, there is limited clinical evidence that supports the use of PPAR agonists against brain-related malignancies. Many clinical trials are still ongoing. One Phase II pilot trial studied rosiglitazone on acromegaly of both macroadenoma and microadenoma in five subjects. Following rosiglitazone treatment (20 mg/day), serum insulin-like growth factor-1 decreased significantly, attributed to inhibition of growth hormone-dependent hepatic synthesis, despite no significant difference in serum growth hormone levels [306]. Two other clinical trials (NCT00612066, NCT00616642) on identifying the potential of rosiglitazone on pituitary tumors had terminated while one is ongoing (NCT03309319). A Phase II clinical trial (NCT01356290) targeting medulloblastoma in children is also currently underway. The primary aim is to evaluate a multidrug anti-angiogenic regimen, which includes fenofibric acid, for medulloblastoma. Minimal conclusions can be derived due to limited clinical data on PPAR agonists for brain-related tumor.

### 13.11. Recurrent/Progressive/Metastatic Cancer

In some clinical trials, PPAR agonists were tested on patients with recurrent or metastatic cancers of different natures. For instance, Robison et al. (2014) employed a multi-drug oral regimen (thalidomide, fenofibrate, celecoxib, low dose of etoposide, and cyclophosphamide) in pediatric patients with varying carcinomas, namely high-grade glioma, ependymoma, low-grade glioma, bone tumors, medulloblastoma/primitive neuroectodermal tumor, leukemia, neuroblastoma, and miscellaneous tumors [307]. Only selected cancers like ependymoma and low-grade glioma yielded favorable treatment response [307]. Two other trials that involved multiple carcinoma strata and used pioglitazone (NCT02133625) and efatutazone (NCT00408434) were completed. The former has no published data, whereas the latter showed one patient (with liposarcoma) had sustained partial response, and 12 patients had stable disease out of the 31 recruited subjects [308]. While the trials showed positive results, multi-modal anti-cancer regimens containing PPAR agonists do not appear remarkably effective against recurrent or advanced cancers. The clinical outcomes of different PPAR agonists in various malignancies are summarized in Table 11.

## 14. Other Diseases

### 14.1. Polycystic Ovarian Syndrome

Polycystic ovarian syndrome (PCOS) is an endocrine disorder that may lead to infertility in females. It is closely linked to chronic diseases like obesity, T2DM, and CVD. In PCOS patients, PPARγ agonists improve insulin sensitivity, hyperinsulinemia, glycemic control, and cardiovascular risk factors like lipid profiles and adiponectin level, but did not affect body weight and body mass index [309,310,311,312,313]. Importantly, pioglitazone also led to favorable changes in hirsutism, hyperandrogenism, and pregnancy occurrence [314,315]. The overall efficacy of TZDs on hormonal and metabolic dysregulation in PCOS females is comparable to metformin [316]. The combined therapy of pioglitazone, metformin with either selective estrogen receptor modulator (clomifene citrate), aromatase inhibitor (letrozole) or antiandrogen (flutamide) also promoted ovulation and lowered excess androgen [317,318]. Although existing studies suffered from small cohort size and short follow-up duration, they suggest that the use of PPARγ agonists can confer promising clinical outcomes in PCOS patients. Clinical trials with larger cohort sizes over a longer term are warranted to elaborate long-term benefits of pioglitazone in PCOS, especially on fertility and pregnancy risk.

### 14.2. Muscular Disorders

Several clinical trials were initiated to explore the clinical prospect of bezafibrate on mitochondrial function in myopathy caused by mitochondrial disorder or neutral lipid storage disease (NCT02398201; EUCTR 2012-002692; NCT01527318). Case studies on two patients with neutral lipid storage disease with myopathy supported the beneficial effects of bezafibrate on tissue lipid accumulation, mitochondrial function, and lipid oxidation, but not on skeletal muscle strength [319]. Additionally, the therapeutic effects of pioglitazone and HPP593 (PPARβ/δ agonist) were also tested in sporadic inclusion body myositis (NCT03440034 and NCT01524406), respectively. While the outcomes from the above trials are not yet available, the findings should shed some light on the safety and clinical efficacy of PPAR agonists in muscular disorders.

### 14.3. Burn Injury

The roles of nuclear receptors are well-implicated in the keratinocyte differentiation and pathological changes of the skin [320]. Conceivably, PPARs, which are members of nuclear receptors, will have unique impact in dermatological conditions. In patients with severe burn injury, a hypermetabolic response, as characterized by elevated cortisol, cytokines, catecholamines, basal energy expenditure, and impaired glucose metabolism, is a common manifestation which can persist up to two years after recovery [321]. Burn patients with hypermetabolism and poor glycemic control had a higher incidence of mortality, infections, and sepsis, but could be rescued with good glucose control [322]. In a Phase II RCT that enrolled pediatric patients with severe burn injury (*n* = 18), fenofibrate significantly improved insulin sensitivity and mitochondrial function [323]. Another larger clinical trial (*n* = 330) is currently on-going to verify the clinical outcomes of fenofibrate as a therapeutic option in thermally-injured patients (NCT02452255).

### 14.4. Miscellaneous Health Conditions

In a proof-of-concept clinical study, fenofibrate showed positive effects in obstructive sleep apnea by decreasing the frequency of oxygen desaturation, apnea, and non-cortical micro-awakenings per hours [324]. Such benefits were not observed with pioglitazone in obstructive sleep apnea, emphasizing the distinct role of each PPAR members [325]. Clinical trials are ongoing for unanticipated diseases, such as sexual dysfunction (NCT00923676) and Huntington’s disease (NCT03515213) using fenofibrate. The trial using rosiglitazone to lower the asymmetric dimethylarginine level in critically ill patients revealed no superiority over placebo [326]. The clinical outcomes of different PPAR agonists in PCOS, muscular disorders, burn injury, and other miscellaneous health complications are summarized in Table 12. It is conceivable that many future trials using PPAR agonists (single, dual, and pan) on other diseases will emerge, which further draw attention to the yet undiscovered potential of PPARs.

## 15. Clinical Prospects of PPAR Agonists and Antagonists

In this review, we attempt to consolidate the clinical evidence from past and ongoing trials featuring PPAR modulators. Although the evidence presented herein is by no means exhaustive, it is undeniable that the clinical implications of PPAR agonists span a wide range of health conditions, ranging from metabolic diseases, chronic inflammatory diseases, infections, autoimmune diseases, neurological, and psychiatric disorders, malignancies, etc. Such an extensive implication of PPAR agonists also signifies the crucial modulatory roles of PPARs in diseases with varying pathogeneses. Coupled with the desirable agonistic effect and safety profile of various PPAR agonists, PPARs are undoubtedly one of the most alluring targets in the medical field.

Based on existing clinical evidence, metabolic and endocrine diseases like T2DM, CVD, dyslipidemia, and MetS remain the mainstay of PPAR clinical research. Given the superior efficacy and high selectivity, pemafibrate is expected to overtake conventional PPARα agonists, in particular, fenofibrate, as the primary fibrate-based lipid-lowering drug in the treatment of dyslipidemia and CVD. Pioglitazone, but not rosiglitazone, will continue to be an important PPARγ agonist for T2DM and CVD prophylaxis among diabetic patients. Despite the setbacks of muraglitazar, tesaglitazar, and aleglitazar, the development of dual- and pan-PPAR agonists in metabolic diseases is gradually gaining momentum, as exemplified by chiglitazar (for T2DM), saroglitazar (for dyslipidemia), and elafibronor (for dyslipidemia). We eagerly await the outcomes of ongoing trials featuring these drugs.

Liver diseases, like NAFLD and PBC, are where dual- and pan-PPAR agonists truly shine. Currently, saroglitazar and elafibranor are in Phase III, whereas lanifibranor is in Phase II for NAFLD. All three drugs show promising results and satisfactory safety profile. Concurrently, elafibranor and saroglitazar are also in the clinical pipeline of PBC. Another drug candidate for PBC is bezafibrate, which is also known to have pan-PPAR agonistic properties. Conceivably, dual- and pan-PPAR agonists are likely to revolutionize the therapeutic paradigm of NAFLD, NASH, and PBC in the future. The feasibility of dual- and pan-PPAR agonists in NAFLD also highlight the potential implications of these drugs in the progression of organ dysfunction caused by ectopic fat deposition in the pancreas, kidneys, and heart.

Pioglitazone is one of, if not the most extensively trialed PPAR agonists. Existing evidence favors the clinical application of pioglitazone in NAFLD, CKD, depressive symptoms, selected autoimmune, and inflammatory diseases, in additional to T2DM and MetS which pioglitazone always thrives in. With the positive efficacy of pioglitazone in these diseases, its clinical utility will likely extend beyond being just an insulin sensitizer. To a lesser extent, PPARα agonists, especially fenofibrate and bezafibrate, also exhibit pleiotropic beneficial effects in dyslipidemia, CVD, NAFLD, and PBC. Therefore, the future prospect of pioglitazone and fibrates remains full of possibilities even after current therapeutic roles (antidiabetic and lipid-lowering agents, respectively) have been replaced by more effective medications. In comparison, the development of PPARβ/δ agonists (e.g., GW501516 and seladelpar) is relatively slow and limited, which is attributable primarily to the pro-oncogenic activity of many members in this pharmacological class. Hence, future synthetic PPARβ/δ agonists should be subjected to a systematic and thorough inspection to ensure the non-existence of carcinogenicity before any human trials.

Thus far, clinical trials of PPAR agonists on neurological and neurodegenerative disorders are mostly negative, whereas those on malignancies yielded mixed results. The negative or conflicting results are indicative of a knowledge gap in our understanding of the roles of PPARs in these diseases. For instance, it is not uncommon for cancer treatment to exhibit efficacy in a context- or cell type-dependent manner. Likewise, the roles of PPARs in the tumor microenvironment may vary, depending on the physiological cues in the tumor microenvironment, which the current PPAR research paradigm often fails to address. As such, until more insights about the roles of PPARs in neurology and malignancy are established, further trials of PPAR agonists in these fields are not recommended.

We note that synthetic ligands that target the same PPAR subtype may not always possess comparable efficacy, safety profiles, and clinical outcomes. Pioglitazone and rosiglitazone are two compelling examples. Rosiglitazone is associated with significant cardiovascular risk, rendering its clinical application highly controversial, whereas pioglitazone continues to show positive activities in many diseases other than T2DM. Such discrepancies can be attributed to the bioavailability, specificity, and pharmacokinetics of the drugs. The inter-drug differences between members of the same class also highlight that the failure of certain investigational PPAR agonists should not stop the creation and development of new agonists. Additionally, while PPAR agonists are widely studied, no clinical evidence is available for PPAR antagonists. Preclinical studies have suggested a role for PPARγ and PPARβ/δ antagonists as anti-obesity and anti-cancer therapy, respectively [327,328]. We speculate that exploring the clinical feasibility of PPARβ/δ antagonists in cancer treatment may result in fruitful outcomes considering the involvement of PPARβ/δ in tumorigenesis processes like oxidative stress homeostasis, differentiation of tumor-associated macrophages, angiogenesis, and epithelial-mesenchymal transition [329,330,331]. Taken together, there is still a great deal of untapped potential in targeting PPARs. Hence, PPAR and their modulators will continue to be a main theme in basic science and clinical research in the years to come.

## 16. Concluding Remarks

PPAR agonists have been clinically tested in a wide range of health conditions with varying degrees of success. Fibrates (PPARα agonists) and TZDs (PPARγ agonists) remain two of the mainstream therapeutic agents for dyslipidemia, T2DM, and CVD prevention. Pemafibrate will likely replace its predecessors (fenofibrate, gemfibrozil, and bezafibrate) in the treatment of dyslipidemia because of its high selectivity, excellent efficacy, and desirable safety profile. Despite the clinical failures of some dual-PPAR agonists due to notable side effects, other multi-target PPAR activators, especially chiglitazar, saroglitazar, elafibranor, and lanifibranor, are quickly picking up the pace and have displayed outstanding efficacy in T2DM, dyslipidemia, NAFLD, and PBC. Pioglitazone is another noteworthy PPAR agonist that shows a promising effect in many non-metabolic diseases, whereas the clinical implication of PPARβ/δ agonists is less well-understood. Side effects are still a major concern in the design and development of new synthetic PPAR ligands. Future PPAR research should explore methods to minimize the off-target effect of PPAR agonists, as well as the potential of PPAR antagonists, as doing so will open a new repertoire for the exploitation of PPAR ligands in medicine.

## Figures and Tables

**Table 1:** Summary of the clinical evidence of PPAR agonists in T2DM.

Disease	Target	Drug Name	Clinical Phase (Sample Size)	Main Findings/Primary Endpoint	Reference/Clinical Trial Identifier
T2DM	Dual PPARα/γ	Muraglitazar	II & III (1477)	▪ Reduced glycemic and lipid parameters were dose-dependently	[52]
			II & III (3725)	▪ Muraglitazar was associated with an excess incidence of the composite end point of death, major adverse cardiovascular events	[65]
		Muraglitazar + sulphonylurea	III (583)	▪ Combined therapy of muraglitazar with sulphonylurea reduced HbA1c, fasting plasma glucose, and triglycerides more effectively than sulphonylurea alone. ▪ Higher rates of congestive heart failure, CVDs, weight gain, and edema in muraglitazar cohort.	[51]
		Muraglitazar + metformin	III (1805)	▪ Muraglitazar plus metformin significantly improved HbA1C, triglyceride, and HDL-C levels	[53]
		Tesaglitazar	II (500)	▪ Reduced fasting plasma glucose dose-dependently ▪ Improved markers of glycemic control, atherogenic dyslipidemia, and measures associated with insulin resistance	[55]
			III-terminated (1707)	▪ Tesaglitazar was comparable to pioglitazone in glycemic control ▪ Tesaglitazar outperformed pioglitazone in improving lipid and lipoprotein levels	[54]
		Tesaglitazar + metformin	III-terminated (555)	▪ Tesaglitazar plus metformin reduced HbA1C, fasting plasma glucose, and insulin levels ▪ The combined therapy also improved triglycerides, HDL-cholesterol, and non-HDL-cholesterol ▪ Higher rate of edema and weight gain in tesaglitazar cohort	[56]
		Tesaglitazar + insulin	III-terminated (392)	▪ Reduced HbA1c, fasting glucose and daily insulin dose ▪ Improved lipid profile ▪ Increased serum creatinine	[57]
		Tesaglitazar + sulphonylurea	III-terminated (568)	▪ Improved glycemic control and lipid profile ▪ Increased serum creatinine dose-dependently	[58]
		Aleglitazar	II (40)	▪ Improved whole-body insulin sensitivity, hepatic insulin resistance index, and total glucose disposal	[59]
			II (332)	▪ Reduced HbA1c dose-dependently ▪ Incidence of edema, hemodilution, and weight gain increased dose-dependently.	[60]
			III-terminated (591)	▪ Improved HbA1c, insulin resistance and lipid variables ▪ Increased incidence of weight gain and hypoglycemia	[61]
		Lobeglitazone	III (173)	▪ Improved glycemic control and lipid profile	[62]
			III (253)	▪ Efficacy of lobeglitazone in glucose-lowering was comparable to pioglitazone	[63]
		MK-0767	NA (8)	▪ Improved adiponectin and lipid profile	[64]
			III-terminated (382)	▪ Primary endpoint: Lipid lowering effectiveness of MK0767 compared to metformin	NCT00543361
			III-terminated (247)	▪ NA	NCT00543010
			III-terminated (129)		NCT00543491
			III-terminated (99)		NCT00543517
			III-terminated (114)		NCT00543738
			III-terminated (610)		NCT00543751
			III-terminated (111)		NCT00543816
			III-terminated (100)		NCT00547274
		Naveglitazar	II-completed	▪ NA	NCT00065312
		ON-5129	II-completed (81)	▪ NA	NCT00335712
			II-completed (105)	▪ Primary endpoint: fasting blood glucose	NCT00212641
		DSP-8658	I-completed (40)	▪ Primary endpoint: Safety assessment	NCT01042106
		Lobeglitazone	IV-ongoing (78)	▪ Primary endpoint: Glycemic target goal achievement rate	NCT02338921
			IV-ongoing (174)	▪ Primary endpoint: Changes of HbA1c	NCT03770052
	Dual-PPAR α/δ	Elafibranor	II (22)	▪ Improved peripheral and hepatic insulin sensitivity ▪ Reduced triglycerides, LDL-cholesterol and liver enzymes	[72]
			II (47)	▪ Reduced triglycerides, LDL-cholesterol, γ-glutamyl transferase, HOMA-IR, glucose, and fructosamine ▪ Increased HDL-cholesterol	[73]
	Pan-PPAR	Indelglitazar	II-completed (108)	▪ Primary endpoint: Changes of fasting plasma glucose, mean plasma glucose during OGTT and insulin	EUCTR 2005-004227-19
			II-completed (500)	▪ Primary endpoint: Fasting plasma glucose	NCT00425919
		Tetradecylthioacetic acid	II-completed (16)	▪ Primary endpoint: Plasma lipids	NCT00605787
		Chiglitazar	III (535)	▪ Reduced HbA1c ▪ Incidences of weight gain, edema, and hypoglycemia were relatively higher in the chiglitazar group	[74]
			III (739)	▪ Chiglitazar was comparable to sitagliptin in lowering HbA1c ▪ Adverse effects were comparable across different groups	[75]
		Lanifibranor	II-ongoing (84)	▪ Primary endpoint: Intrahepatic triglycerides	NCT03459079
	PPARβ/δ	GW677954	II-terminated (1)	▪ Primary endpoint: Changes in fluid related parameters as measured by hematocrit and hemoglobin levels and body weight	NCT00437164
			II-completed (448)	▪ Primary endpoint: Changes in HbA1c	NCT00196989
Diabetic retinopathy	PPARα	Fenofibrate	III (3472)	▪ Reduced diabetic retinopathy progression	[77]
			IV-ongoing (1060)	▪ Primary endpoint: Progression to clinically significant diabetic retinopathy	NCT03439345
		Pemafibrate	III-terminated (15)	▪ Primary endpoint: Diabetic retinopathy worsening or diabetic macular edema development	NCT03345901

**Table 2:** Summary of the clinical evidence of PPAR agonists in CVD.

Target	Drug Name	Clinical Phase (Sample Size)	Main Findings/Primary Endpoint	Reference/Clinical Trial Identifier
Dual PPARα/γ	Aleglitazar	III-terminated (7226)	▪ No effect on lowering CVD risk ▪ Induced side effects like gastrointestinal hemorrhages and renal dysfunction	[78]
		III-terminated (1999)	▪ Reduced HbA1c and blood lipids ▪ Increased incidence of hypoglycemia and muscular events	[79]
		II (332)	▪ Lowered HbA1c dose-dependently ▪ Caused edema, hemodilution, and weight gain	[60]
PPARα	Bezafibrate	NA (1568)	▪ Did not reduce incidence of coronary heart disease and stroke ▪ Reduced incidence of non-fatal coronary events	[83]
		NA (3090)	▪ Reduced mortality risk by 10% ▪ Risk reduction was more prominent in patients with hypertriglyceridemia	[84]
		NA (164)	▪ Improved lipid profile ▪ Reduced fibrinogen ▪ No effect on ultrasound measured arterial disease	[85]
		NA (50)	▪ Reduced fibrinogen ▪ Reduced incidence of angina and left ventricular failure	[86]
	Fenofibrate	NA (9795)	▪ Improved lipid profile ▪ Reduced CVD events	[87]
	Gemfibrozil	NA (4081)	▪ Improved lipid profile ▪ Reduced incidence of coronary heart disease	[88]
	Pemafibrate	III (10000)	▪ Primary endpoint: Number of patients with first occurrence of nonfatal MI, nonfatal ischemic stroke, hospitalization for unstable angina requiring unplanned coronary revascularization, or CV death.	NCT03071692
PPARγ	Pioglitazone	IV (24)	▪ Reduced HbA1c and blood pressure ▪ Enhanced myocardial insulin sensitivity, systolic function and left ventricular diastolic function	[92]
		III (5238)	▪ Reduced CVD events ▪ Reduced risk of recurrent stroke	[93]
		III (5238)	▪ Reduced incidence of major adverse cardiovascular events in long term	[94]
		III (5238)	▪ Reduced all-cause mortality, non-fatal myocardial infarction and stroke	[95]
		III (3876)	▪ Reduced risk of stroke and myocardial infarction among non-diabetic patients with high CVD risk	[96]
		NA (72)	▪ Reduced neointimal volume and inflammatory markers ▪ Increased circulating miRNA-24 and flow-mediated dilation	[97]
		NA (96)	▪ Reduced neointimal hyperplasia in patients with myocardial infarction treated with primary stent implantation	[98]
		NA (97)	▪ Suppressed in-stent neointimal proliferation ▪ Reduced incidence of target lesion revascularization after percutaneous coronary intervention	[99]
		NA (28)	▪ Reduced in-stent stenosis ▪ Reduced leptin and endothelial function	[100]
		III (462)	▪ Pioglitazone was superior to sulphonylurea in slowing the progression of carotid artery intima-media thickness	[101]
		III (543)	▪ Reduced atheroma volume ▪ Improved lipid profile	[102]
		NA (56)	▪ Reduced atherosclerotic plaque inflammation more effectively than sulphonylurea	[103]
		NA (120)	▪ Lowered the recurrence of stroke, but did not reach statistical significance	[104]
		NA (15)	▪ Did not enhance antiplatelet effect	[105]
		III (300)	▪ Efficacy of pioglitazone in cardiovascular function was comparable to sulphonylurea	[106]
	Pioglitazone + metformin	IV (3028)	▪ Combined therapy of pioglitazone with metformin was comparable to combined therapy of sulphonylurea with metformin in preventing CVD events	[107]
	Rosiglitazone	III (193)	▪ No effect on CVD events ▪ Improved glycemic control and cardiometabolic risk profile	[109]
		III (1425)	▪ Reduced carotid intima-media thickness	[110]

**Table 3:** Summary of the clinical evidence of PPAR agonists in dyslipidemia.

Disease	Target	Drug Name	Clinical Phase (Sample Size)	Main Findings/Primary Endpoint	Reference/Clinical Trial Identifier
Dyslipidemia	PPARα	Pemafibrate	III (225)	▪ Reduced triglycerides and liver enzymes ▪ Incidence of adverse drug reaction was lower in the pemafibrate group compared to the fenofibrate group	[118]
			III (526)	▪ Pemafibrate were comparable to high-dose fenofibrate (200 mg/day) and superior to low-dose fenofibrate (100 mg/day) in reducing triglycerides ▪ Adverse effects of pemafibrate was comparable to placebo	[119]
			III (166)	▪ Reduced triglycerides, non-HDL and remnant lipoprotein cholesterol apolipoprotein (Apo) B100, ApoB48, ApoCIII levels, and HOMA-IR. ▪ Increased HDL-cholesterol,ApoA-I, and fibroblast growth factor 21. ▪ Adverse effects of pemafibrate was comparable to placebo	[120]
			III (189)	▪ Decreased triglycerides ▪ Incidence of adverse events was not associated with estimated glomerular filtration rate in dyslipidemic patients with CKD.	[121]
	PPARα, PPARγ	Rosiglitazone and/or fenofibrate	NA (41)	▪ Rosiglitazone alone did not affect triglyceride level.	[122]
	PPARβ/δ	GW501516	II (268)	▪ Increased HDL-cholesterol, APOA-I ▪ Reduced LDL-cholesterol, triglycerides, APOB, free fatty acids	[125]
			IV (13)	▪ Decreased plasma triglycerides, fatty acid, APOB-100, APOB-48, and cholesteryl ester transfer protein activity. ▪ Decreased VLDL-APOB by increasing its fractional catabolism and of APOC-III by decreasing its production rate ▪ Reduced VLDL-to-LDL conversion and LDL-APOB production ▪ Increased HDL-cholesterol, APOA-II, and LpA-I:A-II concentrations by increasing APOA-II and LpA-I:A-II production	[126]
		Seladelpar	II (181)	▪ Reduced APOB-100, LDL-cholesterol, triglycerides, non-HDL-cholesterol, free fatty acids, liver enzymes and CRP ▪ Increased HDL-cholesterol ▪ Reduced number of patients with MetS and higher LDL-cholesterol	[128]
		Seladelpar and/or statins	II (166)	▪ Reduced small and very small LDL particles, and large VLDL ▪ Increased large LDL and HDL ▪ Combined therapy of seladelpar and atorvastatin had complementary effects in improving lipid profile.	[129]
	Dual PPARα/γ	Muraglitazar	II & III-Completed (330)	▪ Primary endpoint: Percent change of triglycerides from baseline	NCT00245388
	Dual PPAR α/δ	Elafibranor	II (94)	▪ Reduced triglycerides and GGT ▪ Increased HDL-cholesterol	[73]
Diabetic dyslipidemia	Dual PPARα/γ	Saroglitazar	III (109)	▪ Reduced triglycerides, LDL, VLDL, total cholesterol, and APOB ▪ No severe adverse event	[130]
			III (302)	▪ Reduced triglycerides, non-HDL-cholesterol, LDL-cholesterol, VLDL-cholesterol, total cholesterol, APOB, and fasting plasma glucose ▪ No severe adverse event	[131]
Familial dysbetalipoproteinemia	PPARα	Fenofibrate or gemfibrozil	NA (146)	▪ Response to fibrate differed based on hypertriglyceridemia subtypes ▪ Hypertriglyceridemia due to lipoprotein lipase deficiency and glycerol kinase deficiency did not respond to fibrate ▪ Palmar xanthomas and hypertriglyceridemia due to APOE resistance responded well to fibrate	[134]
		Bezafibrate	NA (14)	▪ Reduced triglycerides and increased HDL-cholesterol ▪ Changed cholesterol distribution from small- to large-sized LDL and from large- to small-sized HDL	[135]
		Bezafibrate + Statins	NA (15)	▪ Bezafibrate with statin reduced post-fat load triglyceride, APOB and estimated glomerular filtration rate. ▪ Fasting levels of non-HDL-cholesterol, total cholesterol, and HDL-cholesterol were improved with combined therapy.	[136]
X-linked adrenoleukodystrophy	PPARα	Bezafibrate	NA (10)	▪ No reduction in very-long-chain fatty acids	[137]
CPT II and VLCAD deficiencies.	PPARα	Bezafibrate	NA (10)	▪ Reduced LDL, triglycerides, and free fatty acids ▪ No change in palmitate oxidation, fatty acid oxidation and heart rate during exercise did not improve clinical symptoms in patients with CPTII and VLCAD deficiencies	[138]
Familial hypercholesterolemia	PPARβ/δ	Seladelpar	II (13)	▪ Five patients had a ≥ 20% LDL-C decrease from baseline, while another five had a decrease of <15%. ▪ Proprotein convertase subtilisin/kexin type 9 (PCSK9) elevated by seladelpar.	[139]
Familial combined hyperlipidemia	PPARγ	Pioglitazone + lipid-lowering drugs	NA (26)	▪ Increased whole body glucose disposal, myocardial glucose utilization, myocardial blood flow, HDL-cholesterol and adiponectin ▪ Reduced insulin	[140]
			NA (22)	▪ Reduced triglycerides, glucose, and ALT ▪ Increased adiponectin, total and subcutaneous adipose tissues, soleus intracellular lipids	[141]
HIV-associated dyslipidemia and lipodystrophy syndrome	PPARα	Fenofibrate + fish oil	II (100)	▪ Combined therapy of fenofibrate and fish oil significantly reduced triglycerides.	[143]
		Fenofibrate	NA (55)	▪ Reduced triglycerides	[144]
			NA (36)	▪ Reduced triglycerides, APOC-III, total cholesterol, APOB, non-HDL-cholesterol, and triglyceride/APOA1 ratio ▪ Increased HDL-cholesterol, LDL sizes and LDL resistance to oxidation	[145]
			II (99)	▪ Increased HDL-cholesterol ▪ Brachial flow mediated dilation, CRP, IL-6, and D-dimer were unaffected.	[146]
			NA (191)	▪ Decreased triglycerides and total cholesterol ▪ Increased non-HDL-cholesterol ▪ Combined therapy with fenofibrate, niacin, low-saturated-fat diet, exercise, and exercise led to additional benefits on lipid profile compared to all monotherapies.	[147]
		Fenofibrate + Pravastatin	III (174)	▪ Combined therapy of fenofibrate and pravastatin could improve lipid profile and were well-tolerated.	[148]
		Fibrate	NA (245)	▪ Reduced triglycerides and total cholesterol	[149]
			NA (656)	▪ Reduced triglycerides and total cholesterol	[150]
		Bezafibrate	NA (130)	▪ Reduced triglycerides, total, and LDL-cholesterol	[151]
	PPARα, PPARγ	Fenofibrate, pioglitazone	NA (14)	▪ Pioglitazone, but not fenofibrate, improved insulin resistance, blood pressure, and lipid profile.	[152]
	PPARγ	Pioglitazone	III (130)	▪ Increased limb fat deposition, but did not improve lipid profile	[153]
		Rosigltazone	NA (96)	▪ No improvement in lipoatrophy	[155]
			NA (39)	▪ Increased subcutaneous and visceral abdominal fat ▪ Improved insulin sensitivity and adiponectin ▪ Did not affect CRP and flow-mediated vasodilation	[156]
			II (71)	▪ Did not improve carotid intima media thickness, inflammatory markers and endothelial activation markers	[157]
	Pan-PPAR	Tetradecylthioacetic acid	NA (10)	▪ Tetradecylthioacetic acid with diet intervention reduced total cholesterol, LDL cholesterol, triglycerides, LDL/HDL cholesterol ratio, and TNF-α	[159]
Dyslipidemia due to spinal cord injury	PPARα	Fenofibrate	II and III -Completed (23)	▪ Primary endpoint: Triglyceride level compared to baseline after two month fenofibrate treatment	NCT02455336

**Table 4:** Summary of the clinical evidence of PPAR agonists in MetS, obesity, and hypertension.

Disease	Target	Drug Name	Clinical Phase (Sample Size)	Main Findings/Primary Endpoint	Reference/Clinical Trial Identifier
Prediabetes and MetS	Dual PPAR α/δ	Elafibranor	II (47)	▪ Reduced triglycerides, LDL-cholesterol, γ-glutamyl transferase, HOMA-IR, glucose, and fructosamine ▪ Increased HDL-cholesterol	[73]
			II (22)	▪ Improved hepatic and peripheral insulin sensitivity ▪ Reduced triglycerides, LDL-cholesterol, liver enzymes ▪ No activation of PPARs in skeletal muscle, suggesting a liver-targeted action of elafibranor ▪ No safety concern	[72]
Obesity	PPARβ/δ	GW501516	IV (13)	▪ Increased hepatic removal of VLDL and APOA-II production in obese patients	[126]
	PPARγ	Rosiglitazone	IV (28)	▪ Reduced fasting plasma peptide YY3-36 ▪ No effect on fasting ghrelin	[162]
	PPARα	Fenofibrate + metformin	NA (87)	▪ Changes in blood pressure, free fatty acid, BMI and HOMA-IR were comparable to those treated with metformin only ▪ Combined therapy further decreased fasting and postprandial insulin levels	[163]
		Fenofibrate	NA (89)	▪ Reduced body weight, BMI, waist circumference, blood pressure, total cholesterol, LDL-cholesterol, non-HDL-cholesterol, triglyceride and uric acid levels	[164]
Hypertension	PPARγ	Pioglitazone	NA (27)	▪ Reduced systolic and diastolic blood pressure	[167]
			NA (149)	▪ Reduced CRP ▪ Increased 24-h blood pressure profile ▪ Reduced insulin resistance and chronic inflammation	[169]
			III (42)	▪ Increased glucose utilization, norepinephrine spillover response, and baroreflex sensitivity during OGTT ▪ Reduced triglycerides, insulin level and diastolic blood pressure	[170]
			NA (30)	▪ Increased left ventricular diastolic function without causing mass regression ▪ Increased adiponectin and matrix metalloproteinase-2	[171]
		Rosiglitazone	NA (24)	▪ Decreased 24-h systolic and diastolic blood pressure, insulin, plasminogen activator inhibitor-1, CRP, LDL- and HDL-cholesterol ▪ Increased glucose disposal ▪ No effect on plasma glucose	[172]
			NA (16)	▪ No effect on forearm blood flow reactivity ▪ Attenuated free fatty acid elevation upon triglyceride challenge, which preserved endothelium-dependent vasodilation	[173]
	PPARα	Fenofibrate	NA (31)	▪ Did not affect blood pressure in salt-resistant subject ▪ Reduced diastolic and mean arterial blood pressure in salt-sensitive subjects challenged by high-salt diet ▪ Decreased heart rate, plasma renin activity, renal vascular resistance in salt-sensitive, but not salt-resistance subjects	[165]

**Table 5:** Summary of the clinical evidence of PPAR agonists in liver diseases.

Disease	Target	Drug Name	Clinical Phase (Sample Size)	Main Findings/Primary Endpoint	Reference/Clinical Trial Identifier
NAFLD	PPARα	Gemfibrozil	NA (46)	▪ Decreased liver enzymes	[178]
		Fenofibrate	NA (16)	▪ Decreased liver enzymes, hepatocellular ballooning degeneration, and triglycerides	[179]
			NA (27)	▪ Reduced triglycerides, VLDL, and APOB	[180]
		Pemafibrate	II-ongoing (100)	▪ Primary endpoint: Hepatic fat fraction and adverse events	NCT03350165
	PPARγ	Pioglitazone	NA (18)	▪ Improved biochemical and histological features of NASH	[185]
			IV (55)	▪ Reduced liver enzymes and hepatic fat content ▪ Improved histological features of NASH	[186]
			NA (74)	▪ Reduced liver enzymes ▪ Reduced hepatocellular injury, Mallory–Denk bodies and fibrosis	[187]
			III (247)	▪ Reduced liver enzymes ▪ Reduced hepatic steatosis and lobular inflammation	[188]
			IV (101)	▪ Improved biochemical and histological features of NASH	[189]
			NA (10)	▪ Improved ALT levels, mild histological improvement	[190]
		Rosiglitazone	NA (30)	▪ Improved insulin sensitivity ▪ Decreased in liver enzymes and fat content ▪ No change to triglyceride level.	[191]
			II (63)	▪ Improved steatosis, insulin sensitivity and AST/ALT levels	[192]
			II (44)	▪ No improvement in fibrosis, ballooning and steatosis. ▪ Maintained insulin sensitivity and AST/ALT levels.	[193]
			NA (74)	▪ Reduced plasma insulin and improved HOMA-IR score. ▪ Decrease in AST and ALT ▪ Improved NAFLD activity score	[194]
			NA (47)	▪ No change in rate of steatosis and fibrosis ▪ Diet together with exercise was superior to rosiglitazone alone.	[195]
	dual PPAR α/δ	Elafibranor	II (276)	▪ Resolved NASH without fibrosis worsening	[196]
			III-ongoing (2000)	▪ Primary endpoint: Achieving resolution of NASH without worsening of fibrosis	NCT02704403
	Dual-PPAR α/γ	Saroglitazar	III-unknown status (100)	▪ Primary endpoint: Change in NAFLD fibrosis score	NCT02265276
			II-ongoing (106)	▪ Primary endpoint: Percentage change from baseline in serum ALT levels	NCT03061721
			II-ongoing (15)	▪ Primary endpoint: Change in NAFLD activity score with no worsening of fibrosis	NCT03863574
			II-ongoing (60)	▪ Primary endpoint: Change in hepatic fat content	NCT03617263
			II-ongoing (15)	▪ Primary endpoint: Number of participants with adverse events	NCT03639623
		Lobeglitazone	IV (38)	▪ Reduced hepatic fat content ▪ Improved glycemic, liver, and lipid profiles	[197]
	Pan-PPAR	Lanifibranor	II-ongoing (225)	▪ Primary endpoint: Responder analysis based on the improvement of the SAF (steatosis: S, activity: A, and fibrosis: F) activity score	NCT03008070
			II-ongoing (84)	▪ Primary endpoint: Intrahepatic triglyceride level	NCT03459079
PBC	PPARα	Bezafibrate	III (100)	▪ Normalization of bilirubin, liver enzymes, albumin, and plasma triglyceride levels.	[202]
			NA (16)	▪ Decreased levels of liver enzymes	[203]
			NA (66)	▪ Improved biliary enzyme parameters	[204]
			III-ongoing (34)	▪ Primary endpoint: Complete biochemical response	NCT02937012
			III-ongoing (84)	▪ Primary endpoint: Proportion of patients with a reduction in itch intensity of 50% or more	[217]
		Fenofibrate	II (20)	▪ Decrease in liver enzymes, IgM, IL-1, and IL-6 ▪ No significant decrease in bilirubin	[206]
			NA (22)	▪ Decrease in liver enzymes, cholesterol, and TG ▪ No significant effect on serum bilirubin	[208]
			NA (120)	▪ Improvement in ALP, ALT, AST and decompensation, and transplant-free survival	[209]
			II (10)	▪ Decrease in total cholesterol, TG, VLDL, LDL, and liver enzymes	[210]
	PPARβ/δ	Seladelpar	III-ongoing (240)	▪ Primary endpoint: Complete biochemical response	NCT03602560
			II & III-ongoing (356)	▪ Primary endpoint: Adverse events, and treatment emergent adverse events	NCT03301506
			II-ongoing (116)	▪ Primary endpoint: Serum alkaline phosphatase, adverse events	NCT02955602
			II (41)	▪ Normalization of ALP. Risk of grade 3 increase in aminotransferases	[211]
	Dual PPAR α/δ	Elafibranor	II (45)	▪ Primary endpoint: Relative change from baseline in serum alkaline phosphatase	NCT03124108
	Dual PPAR α/γ	Saroglitazar	II-ongoing (36)	▪ Primary endpoint: Improvement in ALP levels	NCT03112681
Hepatitis C	PPARγ	Farglitazar	II (265)	▪ No evidence of antifibrotic activity	[212]
		Pioglitazone	II (5)	▪ No satisfactory viral response.	[213]
			II (40)	▪ Decreased serum HCV RNA.	[214]
			IV (80)	▪ Increased rapid virologic response	[215]

**Table 6:** Summary of the clinical evidence of PPAR agonists in kidney diseases.

Disease	Target	Drug Name	Clinical Phase (Sample Size)	Main Findings/Primary Endpoint	Reference/Clinical Trial Identifier
CKD	PPARγ	Pioglitazone	IV (16)	▪ Decreased the visceral/sub-cutaneous adipose tissue ratio, leptin/adiponectin (L/A) ratio. Increased insulin sensitivity.	[220]
			NA-completed (95)	▪ Primary endpoint: Change in adiponectin and CRP	NCT01301027
			NA (75)	▪ Halved pioglitazone dosage produces similar glycemic effects with reduced adverse effects	[219]
			NA-terminated (36)	▪ Primary endpoint: Change in brachial arterial reactivity	NCT00586261
			IV-ongoing (28)	▪ Primary endpoint: Change in muscle sympathetic nerve activity	NCT03471117
		Rosiglitazone	NA (70)	▪ Improvement to insulin sensitivity, hs-CRP, and von Willebrand Factor (vWF).	[221]
Renal transplant complication	PPARγ	Pioglitazone	NA (48)	▪ Reduced fasting plasma glucose and HbA1c	[223]
			NA (83)	▪ Increase in insulin sensitivity and reduced progression of carotid IMT	[224]
Kidney stone	PPARγ	Pioglitazone	NA (36)	▪ Improvement to features of metabolic syndrome, reduced net acid excretion and increased urine pH	[225]
resistant focal segmental glomerulosclerosis	PPARγ	Pioglitazone	I-completed (21)	▪ Primary endpoint: Safety and tolerance	NCT00193648
Polycystic kidney disease	PPARγ	Pioglitazone	II-ongoing (18)	▪ Primary endpoint: Safety and tolerance	NCT02697617

**Table 7:** Summary of the clinical evidence of PPAR agonists in neurodegenerative diseases and neurological dysfunction.

Disease	Target	Drug Name	Clinical Phase (Sample size)	Main Findings/Primary Endpoint	Reference/Clinical Trial Identifier
Alzheimer′s Disease	PPARγ	Rosiglitazone	II (30)	▪ Better delayed recall and selective attention ▪ Stable plasma amyloid β-42 level at 6^th^ month	[226]
			II (687)	▪ No significant differences of ADAS-Cog at week 24 ▪ APOE ɛ4-negative patients had improved cognitive functions ▪ Dose-dependent improvement of fasting plasma insulin in APOE ɛ4-negative patients	[228]
			II-completed (337)	▪ Primary endpoint: Evaluate the long-term safety and tolerability of rosiglitazone-extended release in subjects with mild to moderate Alzheimer′s disease	EUCTR2004-000985-12
			II (80)	▪ Increased cerebral metabolic rate for glucose ▪ No difference in decrease of whole brain volume ▪ No difference in clinical outcome measures of ADAS-Cog or CIBIC scores ▪ No difference in plasma glucose levels	[227]
			II-completed (40)	▪ Primary endpoint: Frequency of adverse events, safety and tolerability	NCT00381238
			III (639)	▪ No difference in cognition and global function of APOE-∊4-negative subjects ▪ Well-tolerated	[229]
			III (1496; 1485; 1461)	▪ No difference in cognitive function measurements	[230]
		Pioglitazone	III-terminated (3494)	▪ Primary endpoint: Time to diagnosis of mild cognitive impairment due to Alzheimer′s disease	NCT01931566
			II (78)	▪ No increase in oxygen uptake, HbA1c, fasting triglycerides, IL-6 levels ▪ Increased glucose disposal rate ▪ Decreased fasting insulin level and endurance of exercise ▪ No difference in cognitive performances ▪ Significant improvement in ADAS-Cog after endurance exercise	[231]
			II (25)	▪ Well-tolerated ▪ No effect on clinical outcome measures ▪ No change to blood glucose level in non-diabetic subjects	[232]
	PPARα	Gemfibrozil	I-ongoing (72)	▪ Primary endpoint: Safety, microRNA-107 levels, β-amyloid 1-40 and -42 levels	NCT02045056
Amyotrophic Lateral Sclerosis	PPARγ	Pioglitazone	II (219)	▪ Hazard ratio of 1.21, with a 21% increase in the pioglitazone group ▪ No difference in survival, functional rating, quality of life, and slow vital capacity.	[237]
			II (27)	▪ No effect on tau level	[238]
Multiple Sclerosis	PPARγ	Pioglitazone	I (24)	▪ No improvement in disability status score ▪ Reduced grey matter volume and fraction loss	[240]
		CHS-131	II (227)	▪ Dose-dependent reduction in new contrast-enhanced lesions and relapse rates	[241]
Drug-resistant Nocturnal Frontal Lobe Epilepsy	PPARα	Fenofibrate	II (12)	▪ Improved subjective measurements of daily seizure diaries and quality of life ▪ Reduced major events and minor motor events	[242]
Postherpetic Neuralgia	PPARγ	ATx08-001/FK614	II (61)	▪ Primary endpoint: Sum of the pain intensity difference in 6-h and 12-h pain intensity scores	NCT01318226
Friedreich′s Ataxia	PPARγ	Pioglitazone	III (40)	▪ No improvement in neurological functions	[243]

**Table 8:** Summary of the clinical evidence of PPAR agonists in psychiatric disorders.

Disease	Target	Drug Name	Clinical Phase (Sample Size)	Main Findings/Primary Endpoint	Reference/Clinical Trial Identifier
Alcoholism	PPARα	Fenofibrate	II-completed (50)	▪ Primary endpoint: Visual Analog Scale of craving to drink and change from baseline in standard drinks per week	NCT02158273
		Gemfibrozil	II-terminated (3)	▪ Primary endpoint: Drinks per drinking day and percent days abstinent	NCT03539432
	PPARγ	Pioglitazone	II-terminated (16)	▪ Primary endpoint: Alcohol craving in response to the alcohol cue script, lipopolysaccharide challenge, and stress script	NCT01631630
Nicotine dependence/Smoking	PPARα	Gemfibrozil	II (27)	▪ No effect on abstinence, number of smoked cigarettes, and choice for non-nicotinized cigarettes	[245]
			II-completed (16)	▪ Decreased mean carbon dioxide exhaled ▪ Increased mean heaviness of smoking index	NCT02638597
		Fenofibrate	II (38)	▪ No difference in days quit, acute smoking reinforcement, and cue-inducing craving measurements	[246]
	PPARγ	Pioglitazone	I/II (42)	▪ Increased indicators of abuse potential ▪ Reduced measures of craving	[247]
Cocaine dependence	PPARγ	Pioglitazone	I/II (30)	▪ Reducing cocaine craving ▪ Improved fractional anisotropy values of white matter integrity	[248]
Opioid dependence	PPARγ	Pioglitazone	II (32)	▪ No effect on subjective, cognitive, analgesic, and physiological effects of oxycodone ▪ No reduction in drug craving and recreational drug use	[249]
			I (40)	▪ No effect on the prevention of opioid withdrawal symptoms ▪ Increased subjective opiate withdrawal scale score with higher need for rescue medications ▪ Did not reduce proinflammatory cytokines in cerebrospinal fluid or plasma	[250]
Bipolar disorder	PPARγ	Pioglitazone	IV (34)	▪ Decreased depressive symptoms ▪ Improved self-reported depressive symptoms and clinician-rated anxiety scores ▪ Improved cognitive functions and insulin sensitivity	[251]
			IV (38)	▪ No difference in depressive symptoms, response, and remission rates ▪ No change to mania scores, metabolic and inflammatory markers	[256]
			III-ongoing (60)	▪ Primary endpoint: Change in the clinical condition	EUCTR 2014-003803-31
		Rosiglitazone	NA-completed (12)	▪ Decreased depression severity scoring	[252]
	PPARα	Bezafibrate	II-ongoing (30)	▪ Primary endpoint: Change in Montgomery-Åsberg Depression Rating Scale	NCT02481245
Major depressive disorder	PPARγ	Pioglitazone	II-completed (23)	▪ Primary endpoint: Change in Depression Symptom Severity	NCT00671515
			II/III (50)	▪ Improved depression severity score ▪ Led to earlier improvement with better treatment response ▪ Higher remission achieved in pioglitazone group	[253]
			IV (37)	▪ Significant difference in mean decrease of depression scores in insulin resistant subjects ▪ Younger patients with insulin resistance had a greater decline in depression scoring	[254]
Schizophrenia	PPARγ	Pioglitazone	IV (56)	▪ Reduced fasting glucose and high density lipoprotein ▪ Reduced depression symptoms scores, but not cognitive performances ▪ Subjects from China had no improvement in metabolic parameters and psychopathology scorings	[255]
Autism spectrum disorder	PPARγ	Pioglitazone	NA (25)	▪ Reduced aberrant behaviors like irritability, lethargy, stereotypy and hyperactivity	[257]
			II (44)	▪ Reduced irritability, lethargy/social withdrawal, and hyperactivity scores	[258]
			II (25)	▪ Improved behavior scores in global function, social function, irritability, hyperactivity, repetitive behaviors, and anxiety ▪ Decreased IL-6 ▪ Increased IL-10	[259]

**Table 9:** Summary of the clinical evidence of PPAR agonists in autoimmune diseases.

Disease	Target	Drug Name	Clinical Phase (Sample Size)	Main Findings/Primary Endpoint	Reference/Clinical Trial Identifier
Rheumatoid arthritis	PPARγ	Pioglitazone	NA (34)	▪ Reduced CRP and insulin resistance ▪ No effect on swollen or tender joint count and erythrocyte sedimentation rate	[260]
			III (143)	▪ Reduced pulse wave velocity, but not brachial artery flow mediated dilatation and microvascular endothelial function ▪ Reduced disease activity and CRP ▪ Improved lipid profiles	[261]
			NA (49)	▪ Reduced disease activity and CRP	[262]
Systemic Lupus Erythematosus	PPARγ	Pioglitazone	IV (30)	▪ Increased HDL-cholesterol ▪ Reduced insulin, HOMA-IR, CRP, and amyloid A	[264]
			I/II-ongoing (88)	▪ Primary endpoint: vascular function and inflammation	NCT02338999
Autoimmune pulmonary alveolar proteinosis	PPARγ	Pioglitazone	I-ongoing (3)	▪ Primary endpoint: occurrence of adverse events	NCT03231033
Diffuse cutaneous systemic sclerosis	Pan-PPAR	Lanifibranor	III-completed (145)	▪ Primary endpoint: measurement of skin thickness by the Modified Rodnan Skin Score	NCT02503644

**Table 10:** Summary of the clinical evidence of PPAR agonists in inflammatory and infectious diseases.

Disease	Target	Drug Name	Clinical Phase (Sample Size)	Main Findings/Primary Endpoint	Reference/Clinical Trial Identifier
Malaria	PPARγ	Rosiglitazone	I/II (140)	▪ Enhanced parasite clearanceReduced inflammatory response	[265]
			II (30)	▪ Rosiglitazone was well-tolerated in children with uncomplicated malaria	[266]
Ulcerative colitis	PPARγ	Rosiglitazone	II (105)	▪ Increased clinical response and quality of lifeMore patients on rosiglitazone achieved remission	[267]
			NA (14)	▪ Reduced disease activity scoreIncreased adipophilin level	[268]
Asthma	PPARγ	Rosiglitazone	I (34)	▪ Mild reduction of late asthmatic reaction	[270]
		Pioglitazone	IV (68)	▪ No effect on asthma control, airway inflammation, and quality of life	[271]
			II (23)	▪ No effect on asthma control, lung infection, and exhaled nitric oxide	[272]
			NA (34)	▪ No effect on severe asthma	[273]
Plaque psoriasis	PPARγ	Pioglitazone + acitretin	II (41)	▪ Reduced disease severity score	[275]
Endometriosis	PPARγ	Rosiglitazone	II-terminated (3)	▪ Two subjects had less severe symptoms and reduced pain. One patient had no change.	[276]
		Pioglitazone	II and III-withdrawn (20)	▪ Primary endpoint: Peritoneal cytokine level	NCT01184144
Cystic fibrosis	PPARγ	Pioglitazone	NA (20)	▪ Slight reduction of persistent influx of neutrophils in sputumNo effect on sputum inflammatory biomarkers	[277]
			I-completed (24)	▪ Primary endpoint: Sputum biomarkers of lung inflammation and remodeling	NCT00719381
			NA (21)	▪ Primary endpoint: Sputum biomarkers of lung inflammation	NCT00322868
Sepsis	PPARγ	Pioglitazone	I and II (12)	▪ Reduced inflammatory cytokinesPioglitazone was considered safe to critically ill pediatric patients	[278]
Lung inflammation	PPARγ	Pioglitazone	I (18)	▪ No effect on endotoxin-induced lung inflammation	[279]
Lung inflammation due to alcoholism	PPARγ	Pioglitazone	II-ongoing (36)	▪ Primary endpoint: change in phagocytic index of alveolar macrophage	NCT03060772
Chronic granulomatous	PPARγ	Pioglitazone	I and II-ongoing (100)	▪ Primary endpoint: frequency of infections; functional reconstitution of the NADPH oxidase in circulating cells of the peripheral blood	NCT03080480
Gastric phlogosis due to *H. pylori* infection	PPARγ	Pioglitazone	III-ongoing (80)	▪ NA	EUCTR 2005-001218-42
Induced endotoxemia	PPARα	Fenofibrate	NA (36)	▪ No effect on cytokines, chemokines, and acute-phase proteins	[281]

**Table 11:** Summary of the clinical evidence of PPAR agonists in malignancies.

Disease	Target	Drug Name	Clinical Phase (Sample Size)	Main Findings/Primary Endpoint	Reference/Clinical Trial Identifier
Cushing′s disease/ Pituitary tumors	PPARγ	Rosiglitazone Rosiglitazone	II-terminated (2)	▪ Primary endpoint: Number of treatment responders	NCT00612066
			II-terminated (1)	▪ Primary endpoint: Efficacy of rosiglitazone maleate on Cushing disease	NCT00616642
			NA-ongoing (24)	▪ Primary endpoint: Levels of growth hormone, insulin-like-factor 1 (IGF-1), and tumor volume	NCT03309319
Medullablastoma	PPARα	Fenofibrate	II-ongoing (40)	▪ Primary endpoint: Response rate	NCT01356290
Oral leukoplakia	PPARγ	Pioglitazone	II (44)	▪ Induced clinical and/or histologic response in 15 of 21 subjects	[282]
			II-terminated (52)	▪ Primary endpoint: Histologic response and clinical response	NCT00951379
		Rosiglitazone	II-completed (25)	▪ Primary endpoint: Proportion of subjects with complete or partial response in either clinical or histological outcomes	NCT00369174
Oral Cavity / Oropharngeal cancer	PPARγ	Pioglitazone	II-terminated (39)	▪ Primary endpoint: Absolute change in proliferation index (Ki-67) expression	NCT02917629
Anaplastic thyroid carcinoma	PPARγ	Efatutazone/ CS-7017	I (15)	▪ Dose-dependently increased median time to disease progression and survival duration ▪ Increased ANGPTL4 levels and plasma adiponectin	[283]
			II-ongoing (19)	▪ Primary endpoint: Response rate	NCT02152137
PAX8-PPARγ anaplastic thyroid carcinoma	PPARγ	Pioglitazone	II (1)	▪ Reduced acetabular soft tissue metastasis, thyroglobulin, and severe pain upon weight bearing	[284]
Differentiated thyroid carcinoma	PPARγ	Rosiglitazone	II (20)	▪ Five partial responders, three stable disease and 12 progressive disease ▪ No complete or partial responders	[285]
Non-small cell lung carcinoma	PPARγ	Efatutazone/ CS-7017	I (16)	▪ Six patients showed partial response, four with stable disease and six with progressive disease ▪ Well-tolerated	[286]
			II-completed (111)	▪ Primary endpoint: Progression-free survival rate	NCT00806286
			I (14)	▪ Five patients with partial response, four with stable disease, six with progressive disease ▪ Dose-dependently increased of adiponectin level	[287]
			II-completed (90)	▪ Primary endpoint: Proportion of subjects with progression free survival	NCT01101334
		Pioglitazone	II (6)	▪ Reduced proliferation, inflammatory and B-cell survival pathway gene expression ▪ Upregulated complement activation and chemokine signaling gene expression	[288]
			II (92)	▪ No difference in treatment effect ▪ Decreased cellular proliferation	[289]
			II-ongoing (86)	▪ Primary endpoint: Progression-free survival	NCT02852083
Colorectal cancer	PPARγ	Efatutazone/ CS-7017	II-completed (86)	▪ Improved progression-free survival	NCT00986440
			II (100)	▪ No effect on progression-free survival rate ▪ Prolonged overall progression-free survival duration	[290]
Prostate carcinoma	PPARγ	Rosiglitazone	III (105)	▪ No difference in prostate specific antigen doubling time and disease progression	[291]
		Troglitazone	II (41)	▪ One responder, three partial responders and eight non-responders in androgen-dependent prostate cancer group ▪ Four partial responder and 25 non-responders in castration-resistant prostate cancer	[292]
Castration-resistant prostate cancer	PPARγ	Pioglitazone	II (61)	▪ Prostate-specific antigen response in 23 subjects, with 14 with stable disease and 24 non-responders ▪ Reduction or complete regression of bone lesion shown in six of 16 subjects	[293]
	PPARα & PPARγ	Fenofibrate, Pioglitazone, Rosiglitazone	II-terminated (49)	▪ Primary endpoint: Prostate specific antigen doubling time	EUCTR 2006-001398-44
Acute myeloid leukemia	PPARα	Bezafibrate	II (20)	▪ Three subjects had no responses, six subjects had progressive disease ▪ Of the 11 subjects that continued treatment for > 4 weeks without concomitant AML therapy, four had improved hematological scores, with no disease progression in remaining 7 subjects	[294]
			II (18)	▪ Higher toxicities rates with one subject having hematological response	[295]
	PPARγ	Pioglitazone	II-ongoing (94)	▪ Primary endpoint: Overall survival	NCT02942758
Chronic myeloid leukemia	PPARγ	Pioglitazone	I/II-ongoing (100)	▪ Primary endpoint: Cumulative incidence of patients achieving a deep molecular response	NCT02767063
			II-ongoing (26)	▪ Primary endpoint: Number of participants with treatment-related adverse events, treatment free survival after pioglitazone and tyrosine kinase inhibitor discontinuation	NCT02889003
			II-ongoing (31)	▪ Primary endpoint: Treatment-free remission after imatinib discontinuation, number of participants with treatment-related adverse events	NCT02852486
			II-terminated (9)	▪ Primary endpoint: Adverse events, proportion of subjects who achieve and maintain major molecular response	NCT02730195
			II-unknown status (20)	▪ Primary endpoint: Rate of complete molecular response	NCT02687425
			II (24)	▪ Enhanced molecular response	[296]
Cutaneous T-cell lymphoma	PPARγ	Rosiglitazone	II (4)	▪ Reduced skin scores of scaling in two subjects, unchanged in one patient ▪ One subject achieved >50% partial response, while the rest had stable disease ▪ Pruritus alleviated for 3 of 4 subjects ▪ Quality of life unchanged	[297]
Endemic Burkitt′s lymphoma	PPARα	Bezafibrate	II (95)	▪ Disease progression was 29%, 0%, and 0% in low-, intermediate-, and high-dose cohorts respectively ▪ Stable disease/no clinical change at 46%, 71%, and 71% in low-, intermediate-, and high-dose cohorts respectively ▪ Complete clinical response at 39%, 44%, and 68% in low-, intermediate-, and high-dose cohorts respectively	[298]
Multiple myeloma	PPARα	Fenofibrate	II-terminated (6)	▪ Primary endpoint: Response rate	NCT01965834
	PPARγ	Efatutazone	I-terminated (9)	▪ Primary endpoint: Maximum tolerated dose	NCT01504490
			I/II-unknwon status (54)	▪ Primary endpoint: Response rate	NCT01010243
Melanoma	PPARγ	Pioglitazone	I (6)	▪ Progression-free survival ranged from 4–13 months ▪ Four patients with stable disease, one with mixed response with no objective response in radiation field, and one partial responder ▪ Steep decline of melanoma inhibitory activity and improvement of oncological scores in one subject with stable disease ▪ Longer progression-free survival in two subjects with extensive metastatic liver	[299]
Melanoma/Soft Tissue Sarcoma	PPARγ	Pioglitazone	II (40)	▪ Objective response at 11% and disease stabilization > 6 months at 11% for melanoma subjects ▪ Objective response at 19% and disease stabilization > 6 months at 14% for soft-tissue sarcoma subjects ▪ Complete remission in one melanoma subject and three sarcoma subjects ▪ Subjects with normal C-reactive protein levels and subjects with C-reactive levels that decreased by >30% had prolonged progression-free survival	[300]
Skin squamous cell carcinoma	PPARγ	Pioglitazone	II-ongoing (40)	▪ Primary endpoint: Number of squamous cell carcinomas	NCT02347813
Liposarcoma	PPARγ	Troglitazone	II (3)	▪ Induced intracellular lipid accumulation and increased expression levels of PPARγ mRNA	[302]
			II-completed (85)	▪ NA	NCT00003058
		Rosiglitazone	II (12)	▪ Did not induce redifferentiation, reduced proliferation, and upregulation of PPARγ, adipsin, and fatty acid binding protein genes	[303]
Refractory breast cancer	PPARγ	Troglitazone	II (22)	▪ Three patients with stable disease	[304]
Breast cancer	PPARγ	Pioglitazone	I/II (38)	▪ No difference in Ki-67 expression and PPARγ expression ▪ Improved adiponectin and insulin sensitivity	[305]
Acromegaly (macroadenoma and microadenoma)	PPARγ	Rosiglitazone	II (5)	▪ Decreased serum IGF-1 at higher dosage of rosiglitazone (20 mg/day) ▪ No change to growth hormone levels	[306]
Progressive pediatric malignancies	PPARα	Fenofibrate	II (101)	▪ Well-tolerance of antiangiogenic multi-drug treatment ▪ Induced partial response and stable disease in ependymoma and low-grade glioma ▪ Favorable response in miscellaneous central nervous system (CNS) and non-CNS tumors, with nine of 18 having stable disease and five of 18 having partial response ▪ Increased serum TSP-1	[307]
Advanced or metastatic solid tumors	PPARγ	Pioglitazone	I-completed (28)	▪ Primary endpoint: Maximum tolerated dose of pioglitazone and carboplatin	NCT02133625
		Efatutazone/ CS-7017	I (32)	▪ Induced sustained partial response in one patient with myxoid liposarcoma ▪ Twelve of 32 patients had stable disease, with seven of 12 having a ≥81 days stable disease ▪ Well-tolerated	[308]

**Table 12:** Summary of the clinical evidence of PPAR agonists in other health complications.

Disease	Target	Drug Name	Clinical Phase (Sample Size)	Main Findings/Primary Endpoint	Reference/Clinical Trial Identifier
PCOS	PPARγ	Pioglitazone	NA (52)	▪ Increased body weight, BMI and waist-to-hip ratio ▪ Increased insulin sensitivity ▪ Reduced hirsutism, free testoterone, and androstenedione ▪ Increased pregnancy rate	[314]
			NA (30)	▪ Increased insulin sensitivity ▪ Reduced free androgen index	[315]
		Pioglitazone + flutamide + metformin	NA (34)	▪ Reduced androgen ▪ Improved lipid and glycemic control	[317]
		Pioglitazone + metformin + letrozole	I (50)	▪ Increased ovulation and pregnancy rate	[318]
Mitochondrial dysfunction and myopathy	PPARα	Bezafibrate	II-completed (6)	▪ Primary endpoint: respiratory chain enzyme activity	NCT02398201
			II-ongoing (24)	▪ Primary endpoint: Time interval until a predefined decline in muscle performance based on walking test	EUCTR 2012-002692
			NA (2)	▪ Reduced cardiac and muscle fat deposition ▪ Increased fat oxidation	[319]
Neutral Lipid Storage Disease With Myopathy	PPARα	Bezafibrate	IV-completed (6)	▪ Primary endpoints: Mitochondrial function, muscular lipid accumulation, and cardiac function	NCT01527318
Sporadic inclusion body myositis	PPARγ	Pioglitazone	I -ongoing (15)	▪ Primary endpoint: Peroxisome proliferator-activated receptor gamma coactivator 1-alpha target gene expression	NCT03440034
	PPARβ/δ	HPP593	I -terminated (24)	▪ Primary endpoint: Number and severity of adverse events	NCT01524406
Burn injury	PPARα	Fenofibrate	II (21)	▪ Increased glucose uptake, insulin signaling and mitochondrial glucose oxidation	[323]
			II & III-ongoing (330)	▪ Primary endpoint: Glucose metabolism	NCT02452255
Obstructive sleep apnea	PPARα	Fenofibrate	II (34)	▪ Increased oxygen saturation during sleep ▪ Reduced obstructive apneas and non-cortical micro-awakenings per hour	[324]
	PPARγ	Pioglitazone	NA (45)	▪ No effect in quantitative and qualitative sleep measurements	[325]
Sexual dysfunction	PPARα	Fenofibrate	IV-unknown (300)	▪ Primary endpoint: International index of erectile dysfunction (IIEF) in men and Female sexual function index (FSFI) in women	NCT00923676
Huntington′s disease	PPARα	Fenofibrate	II-ongoing (20)	▪ Primary endpoint: Change in peroxisome proliferator-activated receptor gamma coactivator 1-alpha	NCT03515213
Critically ill patients	PPARγ	Rosiglitazone	NA (12)	▪ No effect on asymmetric dimethylarginine in critically ill patients	[326]
